# Surfen, a proteoglycan binding agent, reduces inflammation but inhibits remyelination in murine models of Multiple Sclerosis

**DOI:** 10.1186/s40478-017-0506-9

**Published:** 2018-01-04

**Authors:** Jordan R. Warford, Anna-Claire Lamport, Derek R. Clements, Alicia Malone, Barry E. Kennedy, Youra Kim, Shashi A. Gujar, David W. Hoskin, Alexander S. Easton

**Affiliations:** 1 Department of Pathology, Dalhousie University, Sir Charles Tupper Medical Building, 5850 College Street, PO Box 15000, Halifax, NS B3H 4R2 Canada; 2 Department of Microbiology and Immunology, Dalhousie University, Sir Charles Tupper Medical Building, 5850 College Street, PO Box 15000, Halifax, NS B3H 4R2 Canada

**Keywords:** Surfen, Lysolecithin, Proteoglycan, Experimental autoimmune encephalomyelitis, Multiple sclerosis

## Abstract

Proteoglycans are promising therapeutic targets in Multiple Sclerosis (MS), because they regulate many aspects of the immune response. This was studied using surfen, an agent that binds both heparan sulphate proteoglycans (HSPGs) and chondroitin sulphate proteoglycans (CSPGs). Initial cell culture work on bone marrow derived macrophages (BMDMs) found that surfen reduced concentrations of the chemokines CCL2, CCL4 and CCL5, with reduced messenger (m)RNA expression for Tumor Necrosis Factor, IL-6, IL-1β and inducible nitric oxide synthase. These data were further explored using Experimental Autoimmune Encephalomyelitis (EAE) in mice. Surfen reduced clinical signs during EAE when administered from disease onset, and reduced infiltration by CD4 positive T cells and macrophages into the central nervous system. These mice also showed reduced mRNA expression for the chemokines CCL3 and CCL5, with reduced concentrations of CCL2, CCL3 and CCL5. During EAE, surfen treatment induced a persistent increase in Interleukin (IL)-4 concentrations which may enhance T helper 2 responses. During EAE, surfen treatment reduced mRNA expression for HSPGs (NDST1, agrin, syndecan-4, perlecan, serglycin, syndecan-1) and the CSPG versican. By contrast, surfen increased mRNA expression for the CSPG aggrecan, with no effect on neurocan. During EAE, significant positive correlations were found between mRNA expression and clinical score for syndecan-4, serglycin and syndecan-1 and a significant negative correlation for aggrecan. These correlations were absent in surfen treated mice. Repair in the later stages of MS involves remyelination, which was modeled by injecting lysolecithin (lysophosphatidylcholine, LPC) into mouse corpus callosum to create regions of demyelination. When surfen was injected 2 days after LPC, it delayed remyelination of the lesions, but had no effect when injected 7 days after LPC. The delayed remyelination was associated with local increases in CSPG expression. Therefore surfen suppresses inflammation but inhibits remyelination in these models. A mechanism in common may be increased CSPG expression.

**Electronic supplementary material:**

The online version of this article (10.1186/s40478-017-0506-9) contains supplementary material, which is available to authorized users.

## Introduction

Multiple Sclerosis (MS) is a chronic disabling disease of the Central Nervous System (CNS) that is characterized by plaque formation in the brain and spinal cord parenchyma. These plaques have a prominent inflammatory component in the early stages, associated with myelin stripping from axons (known as demyelination). As the disease progresses, the loss of myelin contributes to atrophy and loss of axons which leads to permanent disability [3]. The pathogenesis of MS is unknown. It may be caused by a persistent failure of myelin formation, either because myelin is biochemically abnormal, or because of damage to myelin forming oligodendrocytes, or be triggered by an auto-antigen that causes autoreactive T cell clones to invade the CNS and trigger demyelination [25]. A puzzling aspect of the disease is the varied ability of plaques to undergo remyelination, which occurs when new myelin sheaths form around previously damaged axons. Many studies have focused on the factors that regulate remyelination, in the hope that restoring the myelin sheath may prevent axonal loss and prevent progression to permanent disability. This progression is untouched by current therapies, which dampen the immune response, but fail to arrest axonal loss. Studies of remyelination have revealed a paradox. While inflammation characterizes the disease, it also plays a role in orchestrating repair and remyelination [7, 18, 21]. Infiltrating macrophages play a role by generating growth factors that stimulate oligodendrocyte precursors to remyelinate axons [13]. However macrophages were also shown to generate chondroitin sulphate proteoglycans (CSPGs) which inhibit maturation of oligodendrocyte precursors, thereby inhibiting remyelination [14].

Proteoglycans (PGs) are macromolecules composed of a protein core covalently linked to glycosaminoglycan (GAG) side chains. GAGs are linear polysaccharides made of repeating disaccharide units and an amino sugar; they comprise heparin, heparan sulphate, hyaluronic acid, chondroitin sulphate, dermatan sulphate and keratan sulphate. The family of heparan sulphate PGs (HSPGs) have heparan sulfate side chains, while chondroitin sulphate PGs (CSPGs) have chondroitin sulphate side chains. The HSPG family includes perlecan, syndecan, serglycin and agrin, while CSPGs include aggrecan, versican, neurocan and brevican. PGs are a major component of the extracellular matrix of the developing CNS where they guide the migration and targeting of neurons to their final anatomic sites [32]. In the adult CNS they are localized in particular sites such as nodes of Ranvier, perineuronal nets and perivascular basement membranes. However their expression in the brain’s extracellular matrix (ECM) increases during many diseases in the process of glial scar formation, as resident astrocytes respond to injury. Reactive astrocytes produce increased amounts of CSPGs, which are inhibitory to axon outgrowth in culture, and play a role in inhibiting axonal regeneration following injury in vivo [5]. In MS autopsy material, in early active plaques there was an accumulation of CSPGs (aggrecan, versican, neurocan) as well as dermatan sulphate PGs at the lesion edge associated with reactive astrocytes, with phagocytosis of PGs by macrophages at the lesion centre [23]. In another study, HSPGs normally associated with the perivascular basement membrane were widely distributed in the parenchyma of active plaques [27]. The role of PGs in the pathogenesis of MS is likely to be multifaceted, given the diverse roles attributed to these molecules, but one effect that they have is to inhibit remyelination. Similar to their ability to inhibit axonal growth and regeneration, a cluster of studies point to the capacity of CSPGs to inhibit remyelination through inhibitory effects on oligodendrocyte precursor cells [14, 17]. However, the effect of PGs is likely to be wider still, since PGs act as binding partners for a variety of factors that regulate immune responses, including cytokines, chemokines and growth factors [22]. Moreover, PGs not only bind to other molecules, but when bound they themselves trigger intracellular signaling events that control cellular responses, including in T cells which are major effector cells in MS [28].

To date there have been relatively few studies that target PGs in an attempt to develop new therapies for MS. A recent study used an inhibitor of CSPG synthesis (fluorosamine) in Experimental Autoimmune Encephalomyelitis (EAE) and lysolecithin (lysophosphatidylcholine, LPC)-induced demyelination and found that fluorosamine improves outcomes in both models. CSPGs alone, or astrocyte generated extracellular matrix containing CSPGs normally inhibits process formation by cultured oligodendrocyte precursors, but this inhibition was partially reversed when the astrocytes laying down the matrix were pre-treated with fluorosamine [12]. The focus of this study is surfen, a compound known to bind the GAG side chains of PGs with high affinity. Surfen was first described as a component of depot insulin, and binds with greatest avidity to heparin, followed (in order) by dermatan sulphate, heparan sulphate and chondroitin sulphate [20]. It is therefore broader in its targeting of PGs than CSPG synthesis inhibitors like fluorosamine, with greater binding to HSPGs than CSPGs. Following on from our previous work in T cells [28], we began by studying the impact of surfen on cultured bone marrow derived macrophages (BMDM) in vitro. Since macrophages are major effector cells in MS, we then went on to study the impact of surfen on two in vivo murine models of MS that respectively utilize EAE and LPC-induced demyelination. These models study complimentary aspects of MS. EAE models the early inflammatory changes seen in MS plaques, and allows detailed study of immunologic aspects of disease. LPC-induced demyelination, which permits the study of remyelination in isolation, mimics the later reparative phases of MS associated with a degree of remyelination, and provides insights into mechanisms of repair.

This study therefore investigates the impact of surfen on activated BMDM which model peripheral macrophages that have infiltrated the CNS during MS, and it goes on to explore the therapeutic potential of surfen in EAE, as well as its effects on remyelination in the LPC model. We report that surfen ameliorates inflammatory responses in EAE, but inhibits remyelination after LPC injection into the corpus callosum. Surfen in EAE has many effects on immune responses, including the expression of chemokines and cytokines, which reflect results obtained in BMDM cultures. The effect of surfen on PG expression has been studied in both animal models, leading to the conclusion that its divergent effects result in part from the increased expression of CSPGs. Surfen therefore inhibits remyelination, not just by binding to PGs, but by triggering increased local expression of CSPGs, which are well known to inhibit remyelination [14, 17]. The beneficial impact of surfen in EAE, and increased CSPG expression, also suggest that CSPGs are protective in the early inflammatory stages of MS, and do not just act as inhibitors of remyelination. These multimodal effects illustrate the complexity of using proteoglyan binding agents as therapies for the different phases of MS.

## Materials and methods

### Reagents and supplies

The peptide fragment containing amino acids 35-55 of Myelin Oligodendrocyte Glycoprotein (MOG_35-55_) was obtained from Stanford Protein and Nucleic Acid Facility (CA). Acetic acid, ammonium sulphamate (H_6_N_2_O_3_S), bovine serum albumin (BSA), brefeldin A from *penicillium*, complete Freund’s adjuvant (CFA) containing *mycobacterium tuberculosis* H37RA (10 mg/ml), concanavalin A, dimethyl sulfoxide (DMSO), chondroitinase ABC, cysteine hydrochloric acid (HCL), 3-(4,5-dimethylthiazol-2-yl)-2,5-diphenyltetrazolium bromide (MTT), eriochrome cyanine, Dulbecco’s Modified Eagle’s Medium (DMEM), 10% buffered formalin, Griess reagent for nitrite, ketoprofen, L-α-Lysophosphatidylcholine (lysolecithin) from egg yolk, phosphate buffered saline (PBS; pH 7.4), pertussis toxin from *Bordetella pertussis,* Roswell Park Memorial Institute 1640 medium (RPMI), heparinase-III, ionomycin, bacterial lipopolysaccharide (LPS) from *E. coli* 0111:B4, papain from papaya latex, phorbol 12-myristate 13-acetate (PMA), sodium azide (NaN_3_), sodium citrate, sodium nitrite (NaNO_2_), sodium phosphate (NaH_2_PO_4_), SIGMA*FAST*® protease inhibitor cocktail tablets, surfen (bis-2-methyl-4-amino-quinolyl-6-carbamide hydrate), and Triton-X-100 were obtained from Sigma-Aldrich Canada (Oakville, ON). Fetal bovine serum (FBS), 10,000 U/ml penicillin/10,000 μg/mL streptomycin solution, 200 mM L-glutamine, 1 M 4-(2-hydroyethyl)-1-piperazineethanesulfonic acid (HEPES) buffer solution, and 0.4% trypan blue dye solution were obtained from Invitrogen Canada (Oakville, ON). Ethylene diamine tetraacetic acid (EDTA) was purchased from EM 46 Industries Inc. (Hawthorne, NY). Anhydrous ethyl alcohol was obtained from Commercial Alcohols (Brampton, ON). Purified functional grade anti-mouse CD28 (clone 37.51), purified functional grade anti-mouse CD16/32 (clone 93), and 7-amino-actinomycin D (7-AAD) were purchased from eBioscience (San Diego, CA). Neutral red was obtained from Fisher Scientific (Nepean, ON). Bio-Rad Protein Assay Dye Reagent, complementary deoxyribonucleic acid (cDNA) iScript, and Arium Total ribonucleic acid (RNA) extraction kits were obtained from Bio-Rad (Mississauga, ON). All cell culture plastics were obtained from Sarstedt Inc. (Montreal, QC) unless otherwise specified.

### Antibodies (Ab)

The following Ab were used for flow cytometry: Phycoerythrin (PE)-conjugated anti-CD25, fluorescein isothiocyanate (FITC)-conjugated anti-CD69, PE-conjugated anti-mouse CD3e (clone eBio500A2), APC-conjugated anti-mouse IFN-γ (clone XMG1.2) were all purchased from eBioscience. Alexa Fluor® 488-anti-mouse CD4 (RM4-5), PE-anti-mouse CD3e (145-2C11), PerCP/Cy5.5-anti-mouse CD8a (53-6.7), APC-anti-mouse CD45 (30-F11), and APC-F4/80 (BM8) were obtained from Biolegend (San Diego, CA). FITC-anti-mouse CD11b (M1/70) was purchased from BD Bioscience (Mississauga, ON).

The following Ab were used for immunofluorescence: an unconjugated primary mouse anti-CSPG polyclonal Ab was obtained from Sigma-Aldrich Canada. An unconjugated rabbit anti-Iba-1 polyclonal Ab was obtained from Wako Chemicals (Wako, TX). Conjugated fluorescent secondary donkey anti-mouse IgG H&L (Alexa Fluor® 555) and donkey anti-rabbit IgG H&L (Alexa Fluor® 488, pre-adsorbed) Abs were obtained from Abcam (Cambridge, UK).

### Bone marrow derived macrophages (BMDM)

#### Cell culture

Mice were killed by cervical dislocation and bone marrow flushed from the tibia and femurs. Red cells were removed by osmotic lysis and the bone marrow cell suspension washed twice with PBS and cultured at a density of 1 × 10^6^ cells/well in 6-well plates in BMDM medium made of 85% RPMI-1640 and 15% L929-conditioned DMEM containing macrophage colony stimulating factor. 6 days later, culture medium and non-adherent cells were removed and fresh BMDM medium added. On day 7 after plating, the purity of BMDMs was 90% based on surface expression of the macrophage marker F4/80 (FACSCalibur, BD Bioscience).

#### Assay conditions

In all experiments, BMDMs were activated with 100 ng/mL of bacterial lipopolysaccharide (LPS), except for the Griess assay and iNOS expression studies which used 500 ng/mL. For nitric oxide assays, flow cytometry, and multiplexed ELISA, BMDMs were seeded in 24-well plates at 2.5 × 10^5^ cells/well in 1 mL of cRPMI medium. Cells were plated at 1 × 10^6^ cells/well in a 6-well plate for qPCR in 3 mL of cRPMI medium. For 7-AAD viability staining, MTT assays and surfen binding, cells were plated in 96-well plates at 1.5 × 10^4^ cells/well in 100 μL cRPMI medium.

#### Protein expression

BMDMs were seeded on 24 well plates in RPMI-1640 and left to adhere for 4 h. One set of wells were left untreated, while others were treated with surfen (5 μM) or vehicle (0.1% DMSO) for 24 h. Culture supernatants were then collected and stored at −80 °C prior to use. Another set of cells were washed briefly in PBS and stimulated with LPS (100 ng/ml) for a further 24 h, at which point the supernatants were collected and stored at −80 °C prior to use. Cytokine levels in the supernatants were assessed using a Bio-Plex Pro™ premixed 8-plex mouse cytokine kit (Bio-Rad, Hercules, CA, USA) that measures the level of the cytokines IL-1β, IL-6, IL-10, TNF and the chemokines KC, CCL2, CCL4 and CCL5, as described above (section 4.3). Data was acquired using Bio-Plex Manager software version 6.1 with five-parameter logistic regression curve fitting (Bio-Rad).

#### Griess assay

After treatment, as outlined in section 1.3, culture supernatants were collected and 100 μL of supernatant combined with 100 μL of Griess reagent in individual wells of a 96-well plate and incubated for 5 min at RT in the dark. Nitric oxide levels for each condition were measured by reading the absorbance at 570 nm using a ELx800 UV universal microplate reader (BioTek Instruments, Winooski, VT) and comparing it with known concentrations of sodium nitrite (Sigma-Aldrich) in PBS using SOFTmax® PRO software (version 4.3; Molecular Devices, Sunnyvale, CA).

#### mRNA expression

After treatment, as outlined above, cells were washed with PBS, and 345 μL of RLT cell lysis buffer (Qiagen) was added to each well of a 6 well plate. RNA was harvested from the lysis buffer using an RNeasy Mini Kit (Qiagen) following manufacturer instructions. The purity and concentration of each RNA sample was determined using a NanoDrop 2000 device (Thermo Fisher Scientific, MA). The purity of the sample was based on the ratio of absorbance at 280 nm over 260 nm (A_280_/A_260_ ratio) in which a value between 1.7 and 2.0 is considered acceptable [25]. Using 500 ng of RNA, cDNA was prepared with an iScript Advanced cDNA synthesis kit (Bio-Rad), as outlined in section 4.4. Transcript levels for individual proteins were determined using an SsoAdvanced Universal SYBR Green Supermix® (Bio-Rad) as outlined in section 4.4. For BMDMs the optimal housekeeping gene was determined to be GAPDH.

#### Viability assays

Viability was assessed by labeling with 7-AAD or MTT. 7-AAD labels non-viable cells by binding to nuclear DNA and is detected by flow cytometry, while the dehydrogenases of viable cells reduce MTT from its yellow tetrazole form to purple formazan, detected by absorbance. In the 7-ADD assay, BMDMs were treated with surfen (1, 2.5, 5, 10, 20 μM) or vehicle (0.1% DMSO) for 24 h. Following treatment, cells were washed with PBS, resuspended in FACS buffer, and labeled with 7- AAD (0.25 μg in 5 μL) for 5 min. Samples were immediately analyzed by flow cytometry. In the MTT assay, following 24 h surfen treatment, 10 μL of MTT solution (5 mg/ml in PBS) was added to each well of a 96-well plate and the BMDMs were incubated for 2 h at 37 °C in a humidified 5% CO_2_ incubator. Following incubation, the plates were centrifuged, supernatants discarded, and 100 μL of 100% DMSO was added to each well. Plates were then shaken for 5 min at 550 g on a Microplate Genie (Montreal Biotech, Montreal, QC). The absorbance at 570 nm was read on an Expert 96 microplate reader (Biochrom ASYS, Cambridge, UK).

#### Surfen binding assay

Unstimulated murine BMDMs were collected and treated with surfen (5, 10, 20 μM) or vehicle (0.1% DMSO) for 2 h. A separate group of BMDMs were pre-treated with heparitinase-III (0.001 U/ml) or chondroitinase ABC (10 U/ml) for 2 h and washed once with PBS before surfen or vehicle was added at the stated doses for an additional 2 h. The cells were washed to remove excess surfen, and fluorescence read at an emission wavelength of 488 nm in a spectrophotometer.

### Mice

C57BL/6 adult female mice (16-20 g, 6-8 weeks of age) were purchased from Charles River Laboratories Canada (Saint Constant, QC) and housed in the Carleton Animal Care Facility (Dalhousie University). Mice were housed for approximately 2 weeks prior to experiments. Experimental procedures conform to ‘Principles of laboratory animal care’ (NIH publication 86-23, revised 1985). All applicable international, national and/or institutional guidelines for the care and use of animals were followed, including guidelines from the Canadian Council on Animal Care. All procedures performed in studies involving animals were in accordance with the ethical standards of the institution at which the studies were conducted and were approved by the Dalhousie University Committee on Laboratory Animals.

### Experimental autoimmune encephalomyelitis (EAE)

#### Induction and surfen treatment

Experimental Autoimmune Encephalomyelitis (EAE) was induced as follows. On day 0, mice were briefly anesthetized with isoflurane (3 L/min) and inoculated subcutaneously (sc) bilaterally at the base of the tail, with a 1:1 mix of MOG_35-55_ and CFA (100 μL each side containing 150 μg MOG_35-55_ and 500 μg *mycobacterium tuberculosis* H37RA) combined with intraperitoneal (ip) pertussis toxin (PTX, 500 ng in 100 μL). The PTX injection was repeated on day 2. Antigen control mice received CFA + PTX but not MOG_35-55_. Mice were scored clinically, based on the assessment of tail and hindlimb paralysis. At day 3, any mice showing lower limb gait disturbances resulting from the immunization procedure were excluded from the study. The scoring scale was 0 - no clinical deficits, 0.5 - partially limp tail, 1 - paralyzed tail, 2 - beginning of walking deficit, 2.5 - one hindlimb paralysed, 3 - both hindlimbs paralysed, 3.5 - hindlimbs paralysed with weak forelimbs, 4 - bilateral hindlimb paralysis and 5 - moribund [24]. At the onset of clinical signs (scores of 0.5 or greater) in mice with EAE, these mice as well as the CFA + PTX group received either vehicle (2.5% DMSO dissolved in PBS [*v*/v], i.p. every other day) or surfen (5 mg/kg, i.p. every other day). Animals were killed on day 21 with a lethal dose of sodium pentobarbital (100 mg/kg, i.p.). CNS tissues were harvested from these animals for subsequent analysis.

#### Extraction of immune cells and flow cytometry

Suspensions of immune cells were obtained from spinal cord and cerebellum. Fresh tissue was dissected, dissociated using a razor blade, and filtered with 70 μm cell strainers to remove debris. Cells were washed in PBS containing 5 mM EDTA, and treated with potassium lysis buffer for 20 min to induce lysis of contaminating red blood cells. Cells were washed twice, blocked with anti-CD16/32 antibody, and incubated with primary conjugated antibodies. Following incubation, cells were washed and fixed with 4% paraformaldehyde prior to flow cytometry acquisition and analysis. Flow cytometry data were collected with a FACSCalibur flow cytometer and CellQuest Pro software (BD Bioscience) or collected with a BD LSRFortessa™ cell analyzer using BD FACSDiva™ software (BD Bioscience). Post-acquisition data analysis was conducted using FCS 6 Express software (DeNovo Software, Los Angeles, CA).5.3 Protein expression.

Protein extracts from EAE and CFA + PTX spinal cord tissue were obtained by homogenization in 500 μL of PBS containing SIGMA*FAST*® protease inhibitor cocktail tablet (2 mM 4-[2-aminoethyl] benzenesulfonyl fluoride, 14 mM E-64, 130 μM bestatin, 1 μM leupeptin, 0.3 μM aprotinin, and 1 mM EDTA). Tissue was homogenized on ice for 1 min using a Pro 200 handheld homogenizer (Pro Scientific Inc., CT). Homogenates were centrifuged at 10000 × g for 10 min, supernatants collected, and protein concentrations determined using a Bradford protein assay. The supernatants were adjusted to a working concentration of 500 μg/mL using PBS buffer containing protease inhibitor and stored at −80 °C prior to use. A Bio-Plex Pro™ premixed 23-plex mouse cytokine kit (from Bio-Rad) was used to measure eotaxin, G-CSF, GM-CSF, Interferon (IFN)-γ, Interleukin (IL)-1α, IL-1β, IL-2, IL-3, IL-4, IL-5, IL-6, IL-9, IL-10, IL-12 (p40), IL-12 (p70), IL-13. IL-17A, KC, CCL2, CCL3, CCL4, CCL5, and Tumor necrosis factor (TNF). Standards were reconstituted with PBS buffer containing protease inhibitor and a 0.1% BSA [*w*/*v*] carrier. A standard curve was constructed using a 4-fold dilution series of beads with a known fluorescence spectrum unique to each cytokine. Magnetic anti-cytokine Ab conjugated beads (50 μL/well) were added to the wells of a 96-well plate and washed twice with 100 μL of assay buffer. Each sample was measured in duplicate wells, by adding 50 μL of undiluted supernatant to each well. The plate was covered and incubated in the dark at room temperature (RT) on a shaker set at 850 rpm for 30 min followed by 3 washes. Next, biotinylated detection Ab was added to each well and incubated in the dark at RT on a shaker set at 850 rpm for 30 min followed by 3 washes. Lastly, streptavidin-PE (50 μL) was added to the wells and incubated in the dark at RT on a shaker set at 850 rpm for 10 min. Lastly, beads were washed 3 times, resuspended in assay buffer and analyzed with the Bio-Plex® 200 Suspension Array System (Bio-Rad). Data was acquired using Bio-Plex Manager software version 6.1 with five-parameter logistic regression curve fitting (Bio-Rad).

#### Messenger RNA (mRNA) expression

Fresh spinal cord tissue was immersed in 10 × the volume of an RNA stabilization reagent (RNAlater, Qiagen, Toronto, ON) for 24 h at 4 °C, and then stored at −20 °C prior to RNA extraction. Tissue was subsequently placed in 2 mL tubes containing 1.5 mm triple-pure zirconium beads (Benchmark Scientific Inc., NJ) and 1 mL of PureZol™ RNA isolation reagent (Bio-Rad). Spinal cord tissues were thoroughly homogenized by placing the bead-filled tubes into a BeadBug microtube homogenizer (Benchmark Scientific, NJ) for 45 s at 4000 rpm. Total RNA was extracted from the homogenized PureZol™ RNA reagent using an Aurum™ Total RNA Fatty and Fibrous Tissue kit (Bio-Rad) following the procedures outlined by the manufacturer. RNA quality was assessed with an Experion RNA StdSens Analysis Kit (Bio-Rad) used in conjunction with an Experion bioanalyzer system. cDNA was prepared with an iScript Advanced cDNA synthesis kit (Bio-Rad). The iScript reaction mix (4 μL) and iScript reverse transcriptase (1 μL) were added to 10 μL of nuclease-free water and 5 μL of input RNA. The reaction was incubated in a Bio-Rad T100™ Thermocycler using the following reaction protocol: 20 min at 46 °C, 1 min at 95 °C, and 5 min at 25 °C. Once synthesized, cDNA was stored at −20 °C for future use. A no-RT control reaction was included in every run to control for genomic DNA carryover by replacing reverse transcriptase volume with nuclease-free water. The transcript level for individual proteins was determined using an SsoAdvanced Universal SYBR Green Supermix® (Bio-Rad). Mouse KiCqStart® SYBR® Green Primers were purchased from Sigma-Aldrich Canada (listed in Additional file 1: Table S1). Each primer set contained a GC content of between 50 and 60%, no secondary structure, melting temperature between 60 °C ± 5 °C, and was between 18 and 24 base pairs long. The optimal annealing temperature for each primer set was determined with a temperature gradient that ranged from 53.0 °C – 65 °C in 2 °C increments using a CFX 96 Touch™ real-time PCR detection system with CFX manager 2.1 software (Bio-Rad). The temperature which produced the lowest quantitation cycle value was chosen as the optimal annealing temperature for that specific primer pair. Next, the linear dynamic range of the samples was established for each primer set by pooling 2 μL of cDNA from each cDNA sample and making a two-fold serial dilution series that ranged from 1/5 to 1/640. Primer efficiency was calculated using the pooled cDNA standard curve and was kept within 90-105% with an R^2^ value >0.980. Subsequent cDNA dilutions were specific to each primer set based on efficiency results. Housekeeping genes were determined by calculating a geNorm expression stability m-value. The m-value represents the maximum amount of heterogeneity across experimental groups that can be tolerated by any given housekeeping gene and must be a value ≤ 1 [26]. The optimal housekeeping genes for spinal cord tissue were GAPDH along with HPRT1 to determine relative expression. The gene B2M was also tested but produced an m-value >1 for spinal cord tissue. PCR amplification and fluorescence detection were performed in triplicate using a CFX 96 Touch™ real-time PCR detection system with CFX manager 2.1 software. PCR cycling conditions were 95 °C for 30 s, followed by 40 cycles of 95 °C for 30 s and the experimentally determined annealing temperature (Additional file 1: Table S1) for 20 s. To confirm that the PCR reaction had produced the appropriate products, a melt curve analysis was conducted with temperatures ranging from 65 to 95 °C in 0.5 °C increments for 5 s each. Results are expressed as relative changes from the housekeeping calibrators and calculated using the 2^-ΔΔCt^ method [15].

### Lysolecithin (lysophosphatidylcholine, LPC) model

#### Injection of LPC into the corpus callosum

Mice were anesthetized with isoflurane (3 L/min) and the head fixed in a stereotactic frame (Model 900, Kopf Instruments, CA). A 1 cm midline incision was made over the scalp. Two holes were drilled in the skull, one 1.2 mm anterior and 1.0 mm left of bregma, the other 1.2 mm anterior and 1.0 mm right of bregma. Using a microinjection unit (Model 5000, Kopf Instruments), 1 μL of 1% LPC [*w*/*v*] dissolved in PBS was injected into the corpus callosum on each side to a depth of 3.7 mm using a 2 μL Hamilton Neuros® 7001 metal syringe (Hamilton Company, NV). To prevent backflow, the needle was kept in place for 30 s before being retracted. The incision was sutured with 5-0 prolene, and the mice allowed to recover. 2 or 7 days later, separate cohorts of mice were reanesthetized with isoflurane, and the midline incision reopened. Through the left burr hole, 1 μL of vehicle (2% DMSO dissolved in PBS) was injected using a Hamilton metal syringe to a depth of 3.7 mm. The right burr hole was used to inject 1 μL of 100 μM surfen in a similar manner to a depth of 3.7 mm. The needle was kept in place for 30 s, retracted and the incision resutured. Sham mice received an injection of either PBS or the needle was inserted but no injection was made, to control for mechanical injury. Different cohorts of mice were killed 2, 7, 14, or 21 days after LPC injection with a lethal dose of sodium pentobarbital (100 mg/kg, i.p.).

#### Myelin staining

Mice were perfuse-fixed through the heart (10 mL PBS followed by 10 mL 10% buffered formalin). The brain was removed and immerse-fixed in formalin for a minimum of 4 days. Paraffin embedded tissue was then sectioned (5 μm). Myelin was stained using eriochrome cyanine with neutral red as counterstain. Briefly, paraffin sections were deparaffinized in 100% xylene, rehydrated in graded ethanols (100%, 95%, 70%), stained with eriochrome cyanine (15 min), washed in tap water (1 min) and differentiated in 0.5% ammonium hydroxide (NH_4_OH) for 5 s. The eriochrome solution consisted of 40 mL of 0.2% eriochrome [*w*/*v*] diluted in 0.5% aq. H_2_SO_4_ [*v*/v] brought to 50 mL with 2% FeCl_3_ [v/v] dissolved in water. Tissue was counterstained with 1% neutral red [w/v] for 2 min, then washed in tap water. Tissue was dehydrated in graded ethanols (70%, 95%, 100%), cleared in 100% xylene and coverslipped using Cytoseal (Stephen’s Scientific, Riverdale, NJ, USA).

#### Immunofluorescence

Formalin fixed paraffin embedded sections were deparaffinized, and sections underwent antigen retrieval in sodium citrate buffer (11 mM sodium citrate, 0.05% Tween 20, pH 6.0) using a decloaking chamber (Biocare Medical, CA). Protein blocking was carried out in 5% donkey serum (Vector Laboratories, CA) dissolved in Tris buffered saline with Tween 20 (TBST) for 1 h at 37 °C. Primary Abs were diluted in TBST and placed directly on each section and incubated for 18 h at RT in the dark using a Simport StainTray™ (Simport Scientific, Beloeil, QC). Next, sections were washed three times with TBST and fluorescent conjugated secondary Ab diluted in TBST and placed directly on the sections for 2 h at RT in the dark. Lastly, sections were washed three times with TBST to remove excess secondary Ab and immediately coverslipped using Vectashield® mounting medium containing the nuclear counterstain DAPI (4,6-diamidino-2-phenylindole, Vector Laboratories, Burlingame, CA).

#### Electron microscopy

A cohort of mice were used following administration of LPC on day 0 and further treatment with surfen or vehicle on day 2. After death on day 7, the brains were removed fresh without transcardial perfusion. The corpus callosum from each side was resected and placed in 3.5% glutaraldehyde and post-fixed for at least 7 days. After fixation, the tissue was washed in Sorensen’s phosphate buffer (0.2 M Na_2_HPO_4_, pH 7.4-7.6) for 2 min. The tissue was then treated with 1% osmium tetroxide (1 h, 4 °C), rinsed in buffer solution (2 min) and passed through a graded series of ethanols (70%, 95%, 100%, 15 min each) followed by treatment with propylene oxide (30 min on mixer). Embedding resin was then added to the tissue (2 h on mixer) and resin added to an embedding mold, pre-warmed in a 70 °C oven. The tissue was transferred to the mold and kept at 70 °C overnight before cooling to RT. Ultrathin sections were cut with a diamond knife on an ultra microtome. The resulting grids were stained with uranyl acetate (8 min), washed three times in 30% ethanol (10 s each wash) and then stained with lead citrate (8 min) and washed three times in distilled water (10 s each wash). The grids were dried, mounted and examined with a Hitachi TT7700 transmission electron microscope. Electron photomicrographs were obtained for different lesions, and stored in image files, coded to blind their identity during image analysis.

#### Image analysis

To evaluate LPC induced lesions in the corpus callosum, slides were scanned with an Aperio AT2 Digital Pathology scanner at 20 × magnification (Leica Biosystems, Wetzlar, Germany). The size of the lesion was determined using Aperio ImageScope software (Leica Biosystems) in which the region of demyelination within the corpus callosum was manually traced with a Wacom Intuos® touch tablet (Wacom Co. Ltd., Kazo, Japan) and the area calculated in mm^2^. Representative photomicrographs of sections that displayed immunoreactivity were captured at 100 × magnification with a Zeiss Axio Imager Z2 fluorescent microscope (Zeiss AG, Oberkochen, Germany). Image quantification was performed using ImageJ software (NIH version). Briefly, preprocessing involved background substraction and application of a noise reduction filter. Next, a global threshold was set to define detection levels across all micrographs. Lastly, fluorescent intensities were quantified in terms of pixel counts translated to area by a scale bar. On electron micrographs, myelination was assessed using the ratio of axon circumference to full circumference (including myelin sheath, if present) of the fiber (G-ratio) using ImageTrak software (written by Dr. Peter Stys; www.ucalgary.ca/styslab/imagetrak). A ratio of 1 represents a completely demyelinated axon, whereas a ratio of 0.5 would be an average ratio of a large myelinated axon. Briefly, lesion electron photomicrographs were imported into the ImageTrak and each axon manually traced with a Wacom Intuos® touch tablet. G-ratios were then automatically calculated.

### Statistics

Where two groups were compared, Student’s t-test and Mann Whitney U test were used to calculate differences between groups with normal and non-Gaussian distributions, respectively. Comparison between three or more groups was performed using a one-way ANOVA with Tukey’s post-hoc analysis. EAE curves were analyzed using a two-way ANOVA with Bonferroni’s post-hoc analysis. All statistics were computed using GraphPad Prism version 7.0 for Macintosh (GraphPad Software, San Diego, CA). *P* < 0.05 was considered significant.

## Results

### Impact of surfen on macrophage cell cultures

**Fig. 1:**
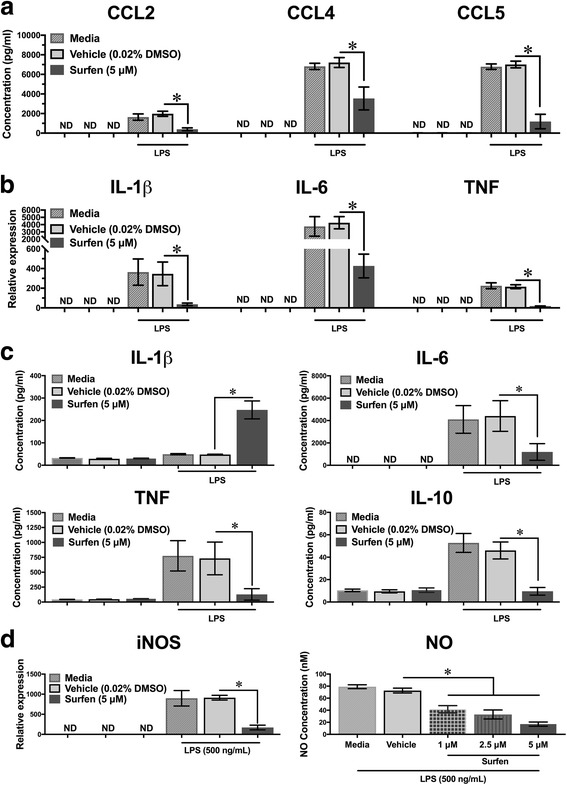
Effects of surfen on factor expression by cultured bone marrow derived macrophages. **a** mRNA expression for selected chemokines. **b** mRNA expression and **c** protein concentration for selected cytokines. **d** mRNA expression for inducible NO synthase (iNOS) and NO production. Data compares cells exposed to media, vehicle or surfen. Bar indicates cells treated with LPS. Significance compares groups linked by cross bars (* = *P* < 0.05). ND = not detectable

Surfen may reduce disease in MS by impacting the function of peripheral macrophages, either in the periphery, or once they have migrated into the CNS parenchyma and undergone local activation. To explore this possibility, cell culture studies were carried out on murine bone marrow derived macrophages (BMDM). 5 μM was the highest dose of surfen that did not reduce numbers or viability of BMDM (significant reductions were noted at 10 and 20 μM, Additional file 2: Figure S1). 5 μM surfen also resulted in significant binding to the cells, as assessed by fluorescence, that was partially inhibited by pre-treatment with heparitinase-III and chondroitinase ABC, enzymes that degrade the GAG side chains to which surfen binds (Additional file 2: Figure S1). Surfen significantly reduced concentrations of the chemokines CCL2, CCL4 and CCL5 released by lipopolysaccharide (LPS)-stimulated BMDM (Fig. 1a). Various cytokines were also studied. In LPS-stimulated BMDM, surfen significantly reduced mRNA expression for IL-1β, IL-6 and TNF (Fig. 1b). Concentrations of IL-6, TNF and IL-10 produced by LPS-stimulated BMDM were significantly reduced by surfen, while, by contrast, the concentration of IL-1β was significantly increased (Fig. 1c). In LPS-stimulated BMDM, surfen significantly reduced the mRNA expression of inducible nitric oxide synthase (iNOS), and induced a dose dependent reduction in NO production (Fig. 1d). These data show that surfen alters the production of key mediators by peripheral macrophages, which may affect their ability to contribute to CNS diseases such as MS, or the animal models we went on to study.

### Surfen ameliorates experimental autoimmune encephalomyelitis (EAE)

**Fig. 2:**
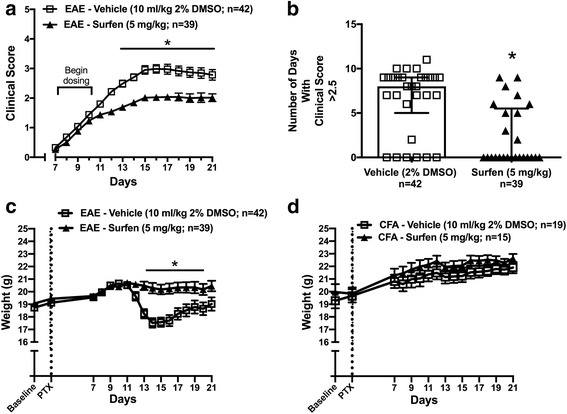
Surfen ameliorates EAE. **a** Clinical scores in mice with EAE, either vehicle treated or surfen treated (dosage and numbers indicated). **b** Number of days spent with clinical scores above 2.5 in each group. **c** Changes in body weight in each group. **d** Changes in body weight in CFA + PTX controls treated with vehicle (CFA - Vehicle) or surfen (CFA - Surfen). Data are shown as mean ± SEM; significant data is marked for surfen versus vehicle, comparing time points (**a**,**c**) or grouped data (**b**); * = *P* < 0.05

Having obtained data from one effector cell of relevance to MS, we went on to study the impact of surfen on EAE, a key model of the inflammatory-immune aspects of the disease. Treatment with surfen from onset of clinical signs during EAE (between 7 and 11 days after induction) resulted in a significant reduction in clinical scores between days 13-21 (Fig. 2a). The mean clinical score for vehicle treated EAE over this period was 2.84 ± 0.05 (mean ± standard error the mean/sem, *n* = 9, averaging the mean for each time point) reducing to 1.96 ± 0.04 (n = 9) in surfen treated mice. A clinical score of ~ 3 represents significant gait impairment with partial hind limb paralysis, while a score of ~ 2 represents mild ataxia. The ordinal scale is limited because the degree of disability is not the same between each interval. Therefore, the number of days spent with a score of ^3^ 2.5 was also calculated, because this reflects substantial gait impairment and paralysis associated with significant spinal cord demyelination [6]. During vehicle treated EAE, mice spent 6.14 ± 0.64 (mean ± sem) days at this level of disability, compared to just 1.87 ± 0.51 days for surfen treated animals (Fig. 2b). There was a significant loss of weight in vehicle treated EAE compared to surfen treated EAE between days 13-19 (Fig. 2c); mean weight over this period in vehicle treated EAE fell to 18.08 ± 0.19 g (*n* = 7) compared to 20.31 ± 0.03 g (n = 7) in the surfen treated group, a reduction in mean weight of 11%. Weights of disease free, CFA + PTX treated control mice remained stable (Fig. 2d) and clinical scores remained at zero in these mice throughout the experimental period. Therefore surfen reduces several disease parameters in the EAE model.

### During EAE, surfen reduces CD4 positive T cell and macrophage infiltration into the CNS

**Fig. 3:**
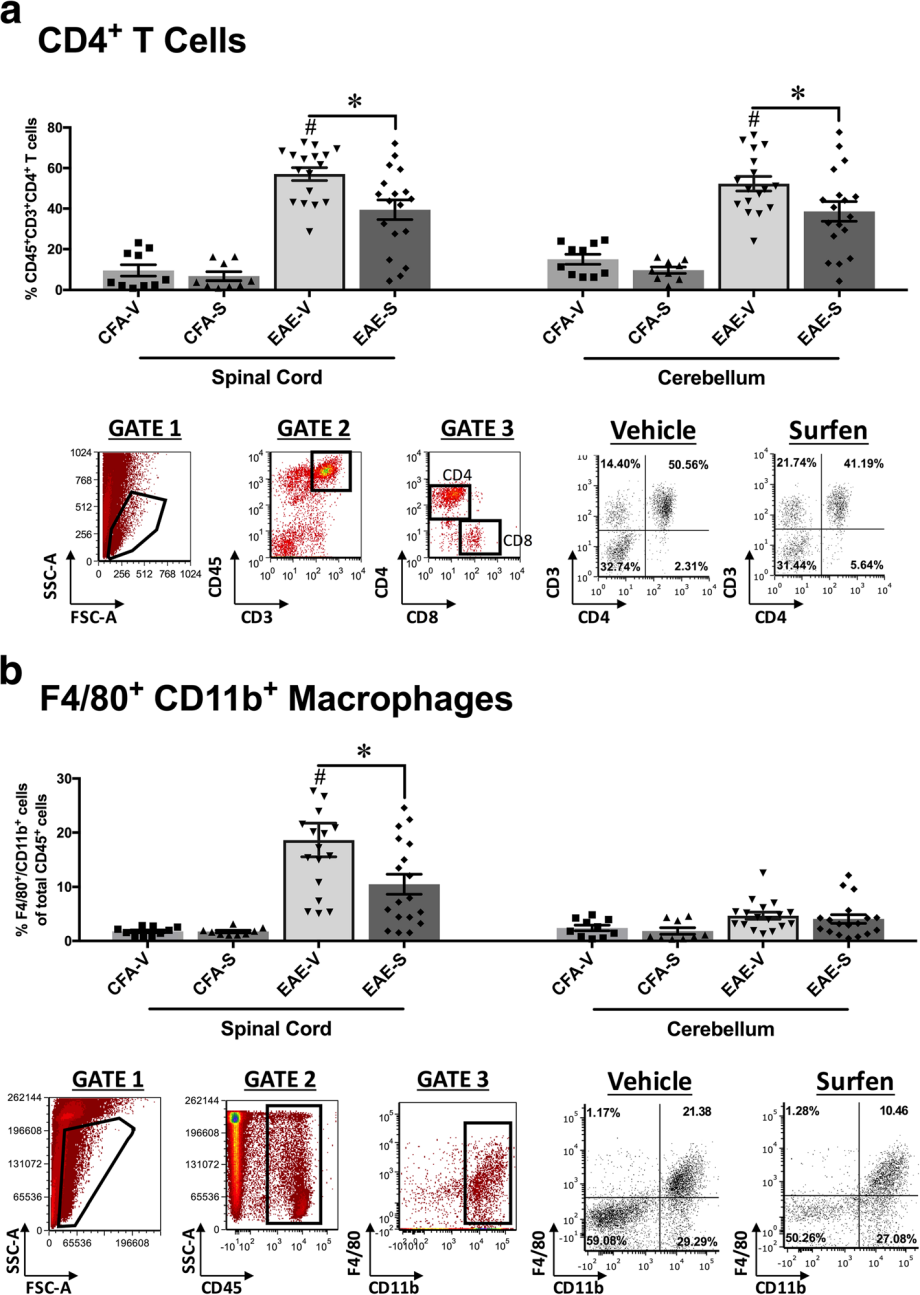
During EAE, surfen reduces cellular infiltration into spinal cord and/or cerebellum. **a** CD4 positive T cells, **b** F4/80, CD11b positive macrophages. Upper panels show % changes in spinal cord and cerebellum for 4 groups. CFA + PTX controls treated with vehicle (CFA-V) or surfen (CFA-S) are compared to EAE treated with vehicle (EAE-V) or surfen (EAE-S). Lower panels illustrate the gating strategy used to calculate the data. Grouped data is shown as mean ± SEM; significant data is marked, comparing vehicle treated EAE with CFA-vehicle control (# = *P* < 0.05) or between groups as indicated by cross bars (* = *P* < 0.05)

Since EAE is driven by infiltrating immune cells, surfen might ameliorate disease by reducing cellular infiltration. T cells and macrophages were identified by flow cytometry in extracts from the spinal cord and cerebellum. Compared to vehicle treated EAE, surfen treatment produced a significant reduction in the percentage of CD4 positive T cells in both spinal cord and cerebellum (Fig. 3a) and of macrophages in spinal cord but not cerebellum (Fig. 3b). In the spinal cord, the percentage of CD8 positive T cells increased during vehicle treated EAE (in CFA + PTX treated controls: 5.02 ± 0.88% (*n* = 10) rising to 14.12 ± 2.12% (*n* = 17) in vehicle treated EAE), but there were no differences between vehicle treated EAE and surfen treated EAE (surfen treated EAE: 12.06 ± 2.34% (*n* = 18)). Representative flow cytometry plots are shown to indicate the gating strategy as well as scatter in surfen treated mice (Fig. 3a,b). In parallel to its effects on the spinal cord, surfen increased the number of cells in peripheral lymph nodes taken from mice with EAE. Surfen also reduced the percentage of proliferating CD4 positive T cells isolated from the lymph nodes and stimulated ex vivo. However surfen had no effect on the percentage of CD4 positive T cells in splenic extracts from mice with EAE (Additional file 3: Figure S2). These data show that surfen reduces infiltration of immune cells into the CNS during EAE.

### During EAE, surfen treatment reduces the expression of the chemokines CCL3 and CCL5, with variable effects on CCL2 and CCL4

**Fig. 4:**
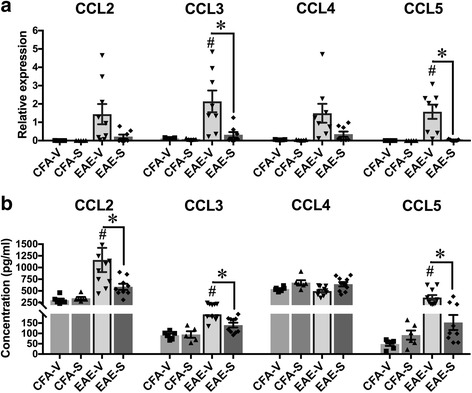
Surfen selectively reduces chemokine expression during EAE. **a** mRNA expression, **b** protein concentration for selected chemokines. CFA + PTX controls treated with vehicle (CFA-V) or surfen (CFA-S) are compared to EAE treated with vehicle (EAE-V) or surfen (EAE-S). Significance is shown as mean ± SEM; significant data are marked, comparing vehicle treated EAE with CFA-vehicle (# = *P* < 0.05) or between groups as indicated by cross bars (* = *P* < 0.05)

One mechanism by which surfen could reduce cellular infiltration into the CNS is to modify local chemokine expression. To explore this possibility, spinal cord extracts were analyzed for levels of the chemokines CCL2, CCL3, CCL4 and CCL5. Relative messenger (m)RNA expression was low in control groups (mice given CFA + PTX and vehicle or CFA + PTX and surfen). Pooled data from these controls showed mean expression for all chemokines of 0.038 ± 0.01 (mean ± sem, *n* = 40). During vehicle treated EAE, mRNA expression for CCL3 and CCL5 was significantly increased (Fig. 4a). Mean expression varied between 1.44 (CCL2) and 2.14 (CCL3). Surfen treatment during EAE resulted in a significant reduction in mRNA expression for CCL3 and CCL5. Mean expression for CCL2 and CCL4 reduced to between 0.21 (CCL2) and 0.35 (CCL4), which was not significant (Fig. 4a). When protein concentrations were measured, significant increases occurred during vehicle treated EAE for CCL2, CCL3 and CCL5. Levels of these chemokines were significantly reduced by surfen treatment during EAE (Fig. 4b). Concentrations of CCL4 were unaltered. These reductions in chemokine expression provide a partial explanation for the reduced cellular infiltration induced by surfen treatment during EAE.

### Surfen treatment modifies the expression of T cell regulators in EAE

**Fig. 5:**
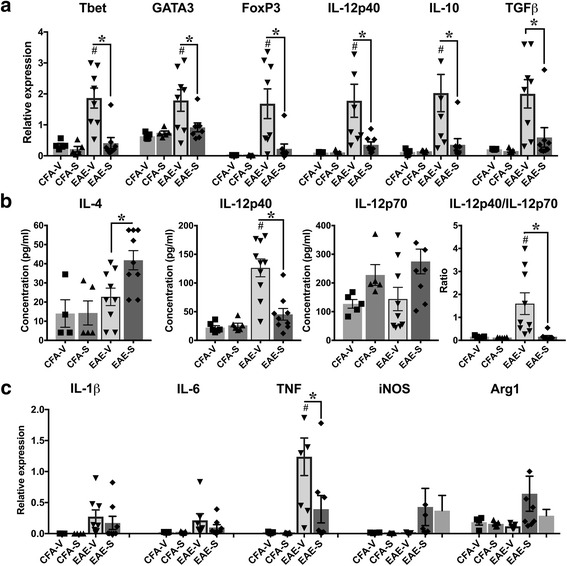
Effect of surfen on T cell regulators during EAE. **a,c** mRNA expression for indicated regulators (see Results for details), **b** protein concentrations. Data compare CFA + PTX controls treated with vehicle (CFA-V) or surfen (CFA-S) to EAE treated with vehicle (EAE-V) or surfen (EAE-S). Significance is shown as mean ± SEM; significant data are marked, comparing vehicle treated EAE with CFA-vehicle (# = *P* < 0.05) or between groups as indicated by cross bars (* = *P* < 0.05)

As well as an impact on cellular infiltration, surfen may reduce disease by direct effects on T cell function within the CNS. To explore this possibility, proteins involved in different T cell responses were studied during EAE in surfen treated mice. mRNA expression of the T helper (Th) 1 transcription factor Tbet, Th2 transcription factor Gata3 and T regulatory (Treg) transcription factor FoxP3 were significantly increased during vehicle treated EAE and significantly reduced in surfen treated EAE (Fig. 5a). There were significant positive correlations between mRNA expression and clinical score for these transcription factors. For Tbet, the Spearman correlation coefficient (R) was 0.73 (*p* = 0.01), for GATA3 it was 0.68 (*p* = 0.003) and for FoxP3 it was 0.79 (*p* = 0.003). mRNA expression of the transcription factor RORγT which regulates Th17 responses was unaltered (data not shown). mRNA expression of the Th1 cytokine IL-12p40 and Th2/Treg cytokine IL-10 was also significantly increased during vehicle treated EAE, while the increase in mean expression of the Treg cytokine TGF-β was not significant. Expression for all three cytokines was significantly reduced in surfen treated EAE (Fig. 5a). In spite of reductions in mRNA for the Th2 factors Gata3 and IL-10, surfen treatment during EAE induced a significant increase in concentration of the Th2 cytokine IL-4 (Fig. 5b) but had no effect on the Th2 cytokines IL-5, IL-10 and IL-13 (data not shown). The reduction in mRNA expression for IL-12p40 was paralleled by significant reductions in protein concentration in surfen treated EAE (Fig. 5b). There was a non-significant trend for surfen to increase the concentration of IL12p70, with an increased mean in surfen treated CFA + PTX controls and surfen treated EAE. This is reflected in the significant reduction in the p40/p70 ratio during surfen treated EAE (Fig. 5b). During EAE, there was a significant increase in mRNA expression for Tumor Necrosis Factor (TNF), but not for Interleukin (IL)-1β or IL-6. The increase in TNF expression was suppressed by surfen treatment during EAE (Fig. 5c). Surfen did not alter protein levels for these cytokines (IL-1β: 156.5 ± 15.5 pg/ml (vehicle treated EAE, *n* = 10) v. 161.7 ± 14.94 pg/ml (surfen treated EAE, n = 10); IL-6: 91.17 ± 16.30 pg/ml (vehicle treated EAE, *n* = 9) v. 97.49 ± 9.93 pg/ml (surfen treated EAE, *n* = 10); TNF: 1046 ± 156.4 pg/ml (vehicle treated EAE, n = 10) v. 1302 ± 159.3 pg/ml (surfen treated EAE, n = 10). Surfen also had no effect on concentrations of IL-17A, IL-2 or Interferon (IFN)-γ (data not shown). mRNA expression for inducible nitric oxide synthase (iNOS) and arginase-1 were not significantly increased during EAE, and surfen had no effect on their expression (Fig. 5c). These data suggest that surfen prolongs the Th2 response during EAE, which may contribute to disease suppression.

### Surfen modulates proteoglycan (PG) expression during EAE

**Fig. 6:**
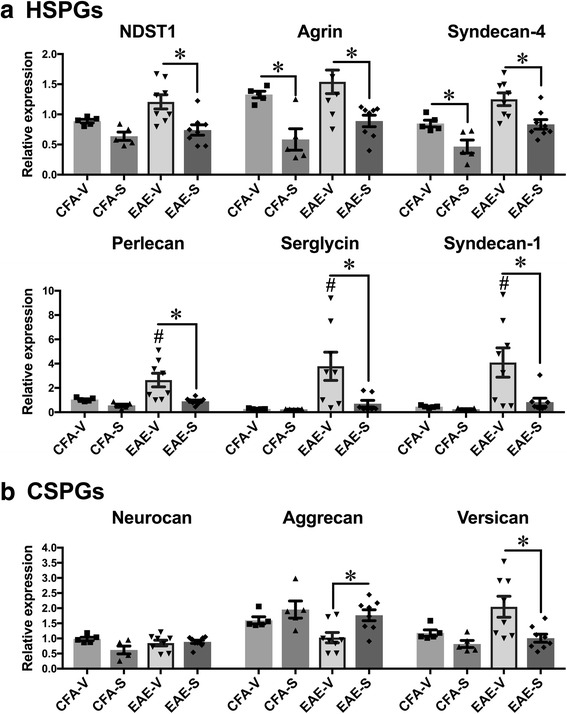
Effect of surfen on proteoglycan mRNA expression during EAE. **a** Expression of HSPGs as indicated. **b** Expression of CSPGs as indicated. Data compare CFA + PTX controls treated with vehicle (CFA-V) or surfen (CFA-S) to EAE treated with vehicle (EAE-V) or surfen (EAE-S). Significance is shown as mean ± SEM; significant data are marked, comparing vehicle treated EAE with CFA-vehicle (# = *P* < 0.05) or between groups as indicated by cross bars (* = *P* < 0.05)

**Fig. 7:**
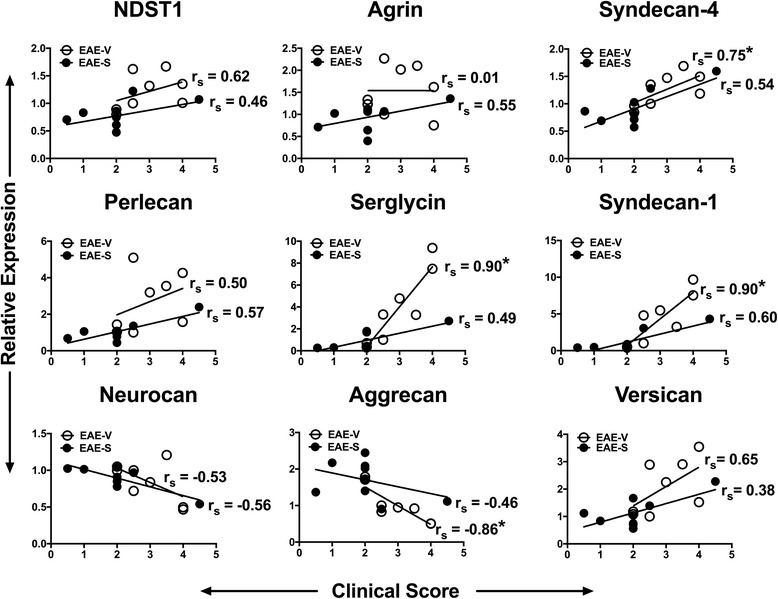
Correlations between proteoglycan mRNA expression and clinical score during EAE. The data points relate mRNA expression to clinical score for individual mice with vehicle treated EAE (open circles) or surfen treated EAE (filled circles). Spearmann’s correlation coefficient (r_s_) for each group is shown. * indicates significance (*P* < 0.05)

PG expression within the CNS is known to change within MS plaques and when modulated, it can impact disease severity during EAE [12, 27]. Therefore, surfen may influence EAE by modulating PG expression. The data show that surfen reduced mRNA expression of a number of PGs during EAE. Significant increases in mRNA expression occurred during vehicle treated EAE for 3 of the 6 heparan sulphate proteoglycans (HSPGs) studied: perlecan, serglycin and syndecan-1. The remaining 3 HSPGs were unaltered (syndecan-4, N-deacetylase/N-sulfotransferase-1 (NDST1) and agrin). However, compared to vehicle treated EAE, mice treated with surfen during EAE showed significant reductions in expression for all 6 HSPGs (Fig. 6a). The chondroitin sulphate proteoglycans (CSPGs) neurocan, aggrecan and versican were also studied. While there were no significant changes in expression during vehicle treated EAE, in surfen treated mice the level of aggrecan expression was significantly increased, matching levels in both CFA + PTX controls. By contrast, versican levels were significantly reduced, following the pattern noted for the HSPGs. Neurocan levels remained unchanged as a result of surfen treatment (Fig. 6b). The mRNA expression for each PG was also related to clinical scores both during vehicle treated EAE and surfen treated EAE (Fig. 7). In vehicle treated EAE, there were significant positive correlations between PG expression and clinical score for serglycin, syndecan-1 and syndecan-4. There was a significant negative correlation for aggrecan. Significant correlations were absent in surfen treated EAE, which may indicate that surfen ameliorates disease by lowering HSPG expression and increasing expression of the CSPG aggrecan.

### Surfen inhibits remyelination in the LPC model

**Fig. 8:**
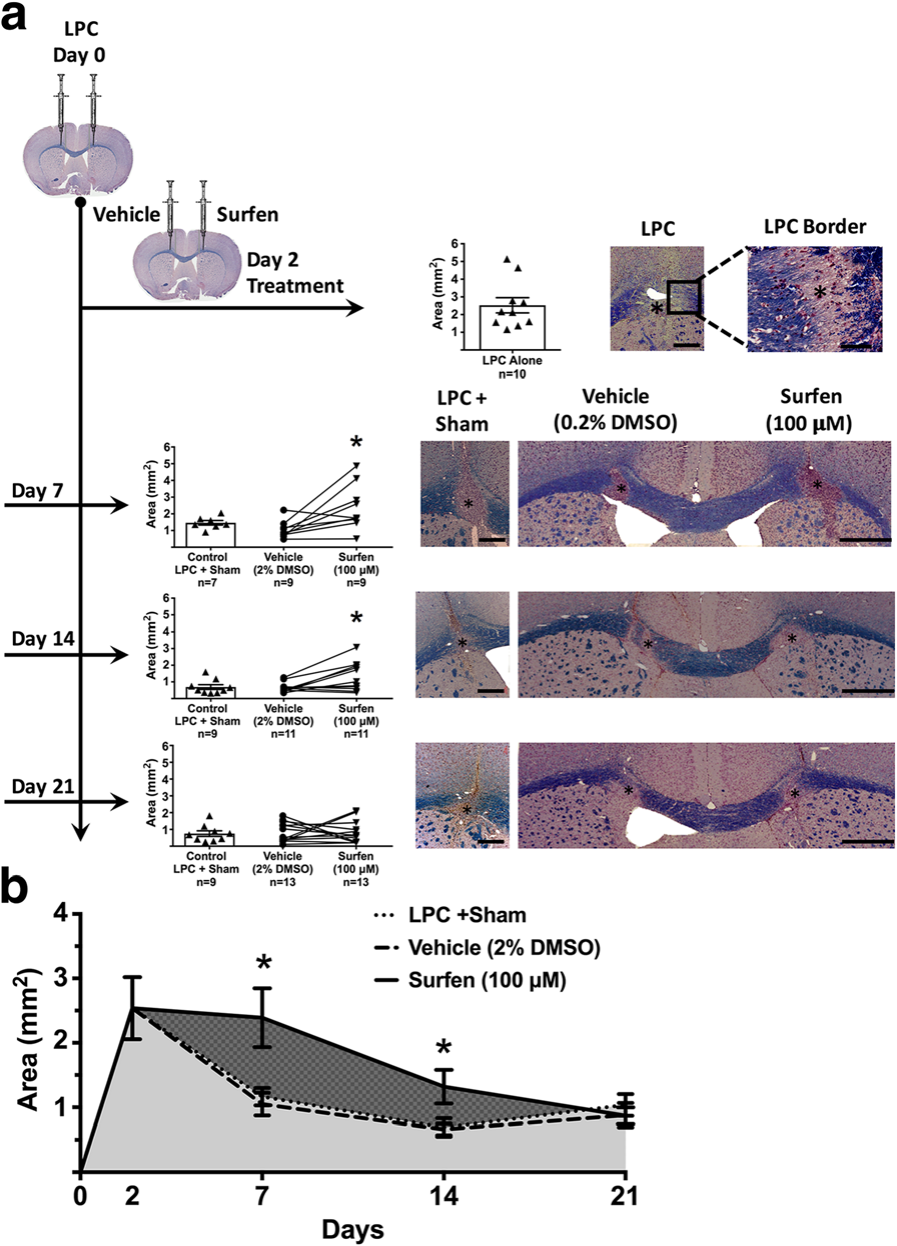
Surfen injected 2 days after lysolecithin (lysophosphatidylcholine, LPC) delays remyelination of lesions in the corpus callosum. Surfen is administered 2 days after LPC injection. **a** Left sided linear graphic shows treatment schedule, while right sided panels show data for each time point, as well as representative images of lesions in the corpus callosum. **b** The area of the lesions is plotted against time from LPC injection for groups indicated. Data is shown as mean ± SEM; significant differences between matched surfen and vehicle injected groups are indicated (* = *P* < 0.05); Scale bars: LPC, LPC + Sham = 300 μm; LPC border = 100 μm, Vehicle/Surfen = 500 μm

**Fig. 9:**
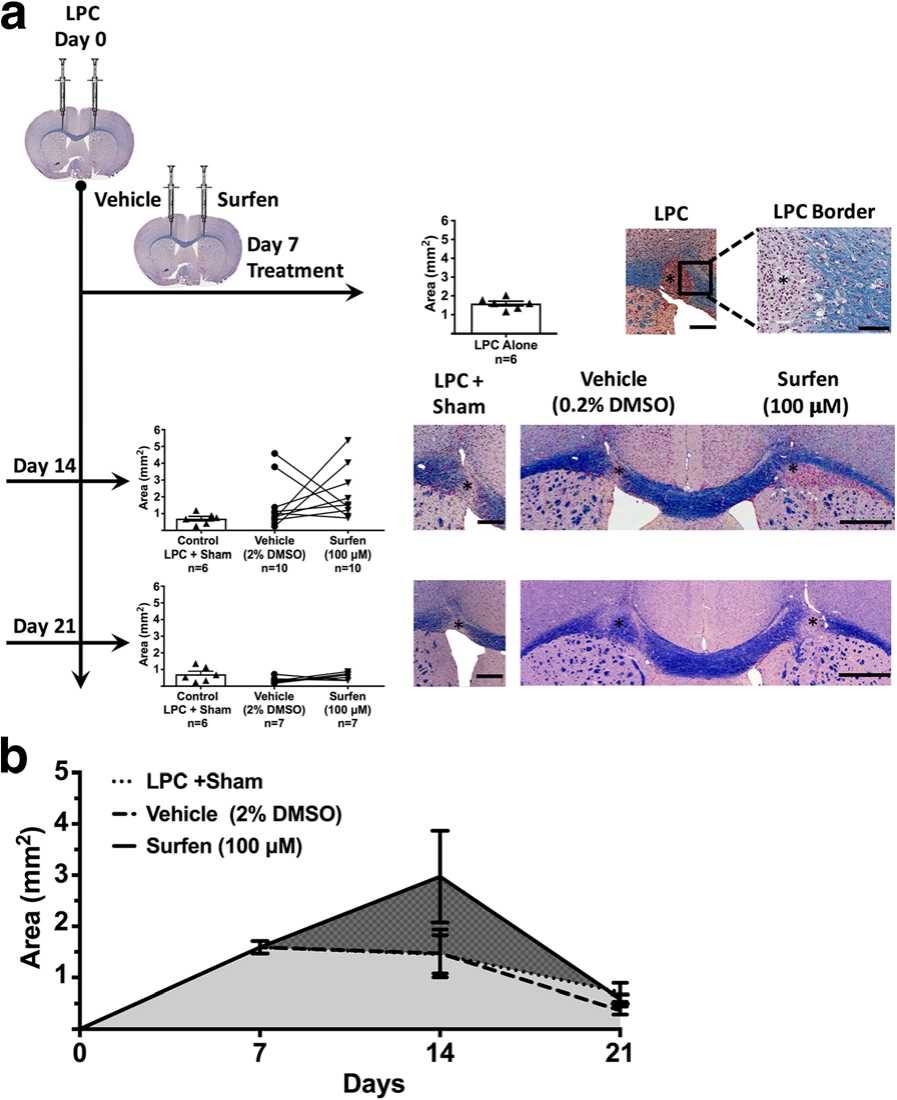
Surfen has no significant effect on lesion size when administered 7 days after LPC injection. **a** Left sided linear graphic shows treatment schedule, while right sided panels show data for each time point, as well as representative images of lesions in the corpus callosum. **b** The area of the lesions is plotted against time from LPC injection for groups indicated. Data is shown as mean ± SEM; Scale bars: LPC, LPC + Sham = 300 μm; LPC border = 100 μm, Vehicle/Surfen = 500 μm

**Fig. 10:**
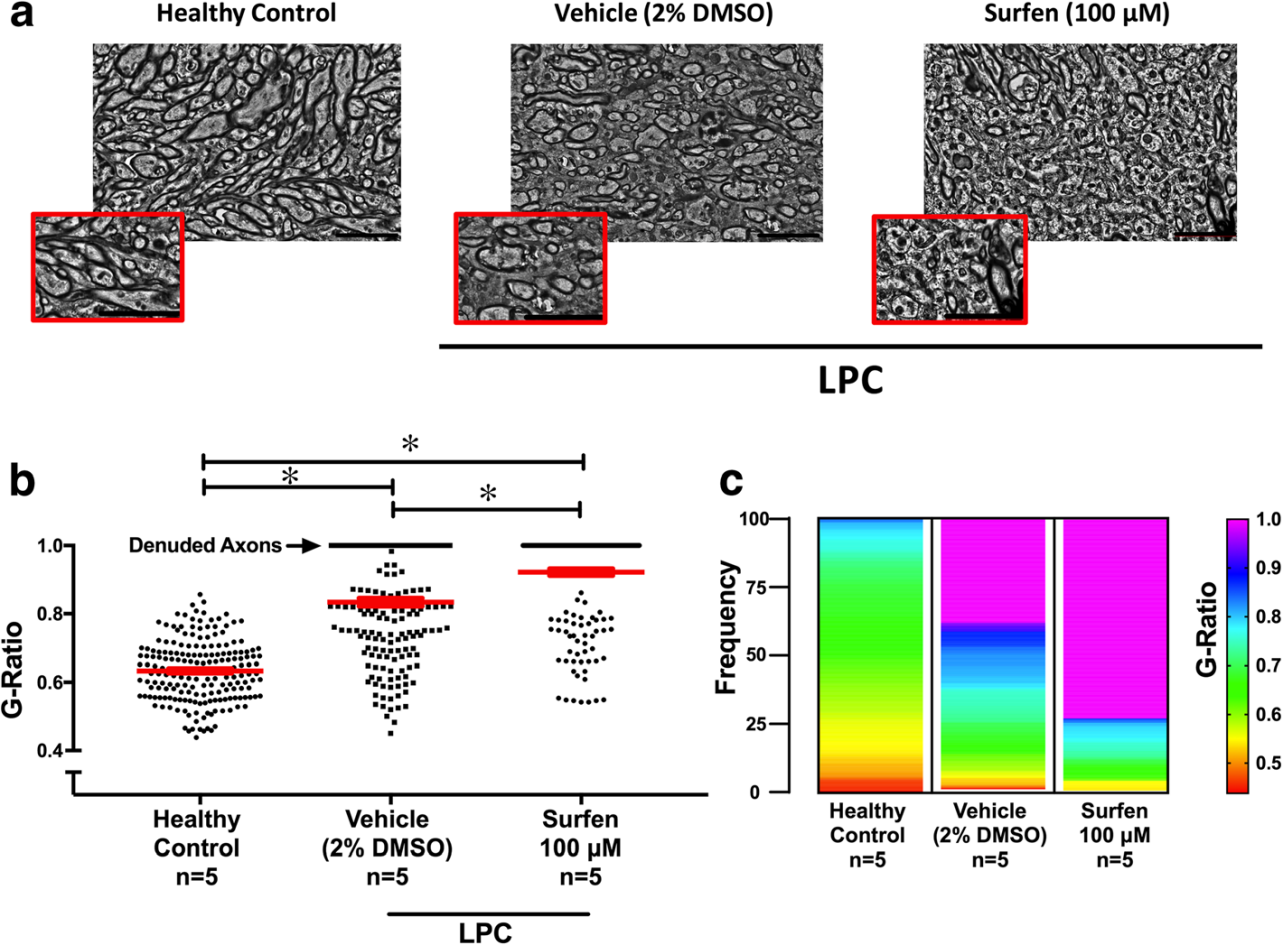
Surfen injected 2 days after LPC prevents remyelination at day 7. **a** Representative electron micrographs for the normal corpus callosum and day 7 lesions in mice injected with vehicle or surfen 2 days after LPC. Scale bars = 50 μm. Higher magnification insets are also included with each micrograph. **b** Summary data for G-ratios. Left panel shows individual G ratios for axons in each group, with superimposed mean ± SEM. Significant differences between groups are indicated above linking cross bars (* = *P* < 0.05). **c** Frequency distribution plot using pseudocolor for each group. *N* indicates number of mice used to obtain the data

**Fig. 11:**
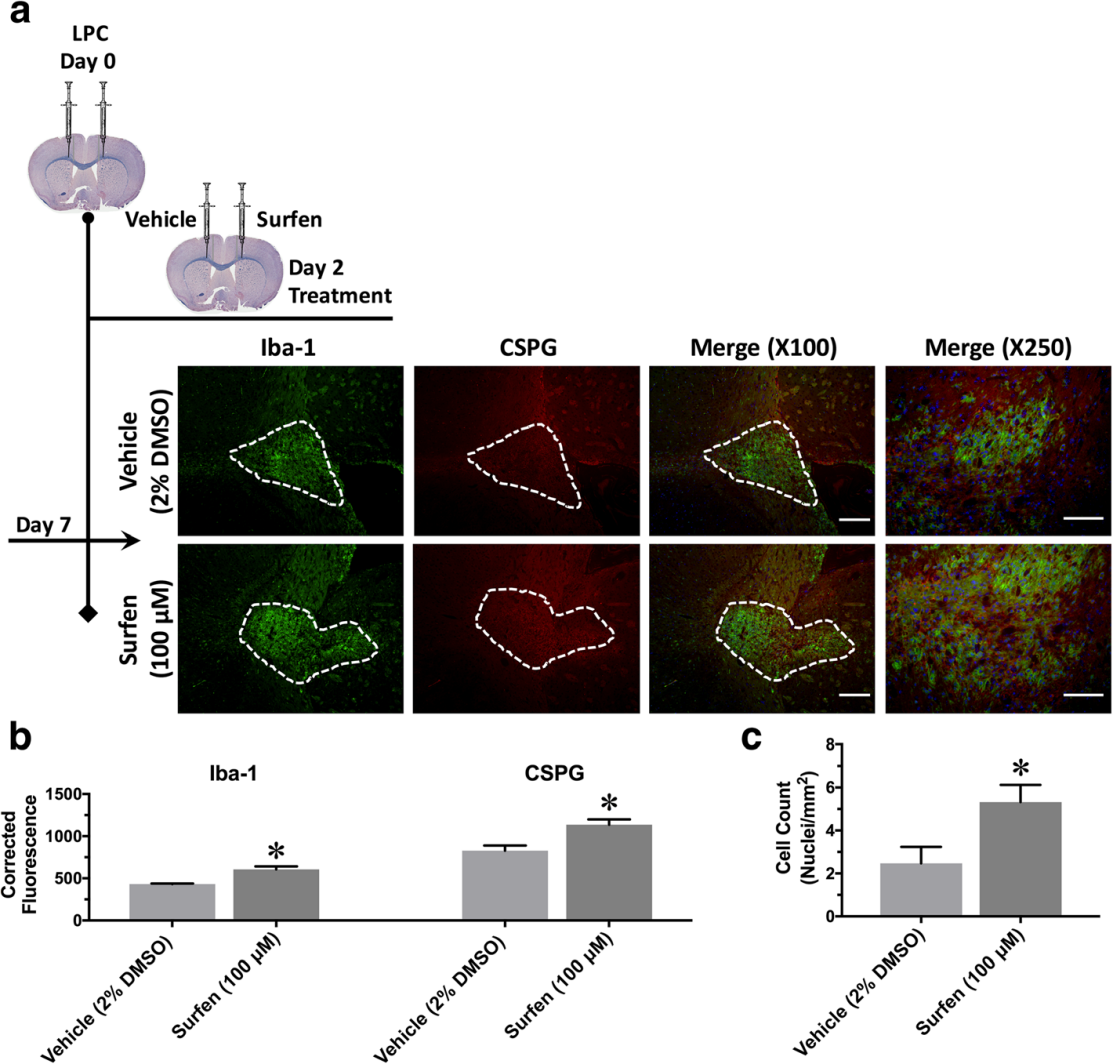
Surfen injected 2 days after LPC increases local expression of CSPG and Iba-1 with increased cell counts at day 7. **a** Left sided linear graphic shows treatment schedule. Representative immunofluorescence images are shown for Iba-1,CSPGexpression in groups indicated. Merge image (×100 magnification)shows no overlap for Iba-1 and CSPG. Higher magnification merge image (×250) was used to visualize DAPI nuclear staining for cell counts. **b** Summary data for Iba-1 and CSPG expression. c. Summary data for cell counts. *N* = 3 mice per group significance compares surfen with vehicle (* = *P* < 0.05); Scale bars = 100 μm

The later phases of MS have been modeled by injecting lysolecithin (lysophosphatidylcholine, LPC) into the corpus callosum to create a discrete region of demyelination, similar to a demyelinated MS plaque. LPC is generated by phospholipase A2, and can both act as a detergent, solubilizing lipid rich membranes such as myelin, and as a toxin to myelin forming oligodendrocytes [9]. It has been injected into various white matter tracts in rodents, including the dorsal columns of the spinal cord [14], the caudal cerebellar peduncle [29] and corpus callosum as here [16] to create a demyelinated focus that then undergoes progressive remyelination, which has been noted in certain MS plaques, and mimics the processes of repair. The LPC model was used to determine if surfen would promote or inhibit remyelination, and potentially promote (or inhibit) repair during MS. When surfen was co-injected with LPC, lesion size was unaltered 7 days later (Additional file 4: Figure S3). This indicates that surfen does not interfere with the demyelinating action of LPC. Demyelination is maximal 2 days after LPC injection, when lesion size is at its peak. Subsequent remyelination proceeds rapidly between day 2 and 7; between day 7 and 21 there is little further reduction in lesion size (Fig. 8b, sham data). When surfen was injected into the lesion at day 2 it impeded remyelination, so that lesion size was virtually unchanged between day 2 and day 7. Remyelination did occur in the surfen injected group, but was considerably delayed. By day 14 in this group, lesion size was still significantly greater than the sham group, however the two groups converged at day 21 (Fig. 8b). When surfen was injected into the lesion at day 7, that is after most remyelination had occurred, then it had no significant effect on subsequent remyelination (Fig. 9). At day 14, there was a tendency for the lesion to increase in size after surfen injection at day 7, but the surfen data is too variable to show a significant increase (mean lesion size in the surfen group was 2.97 ± 0.90 mm^2^ (*n* = 10) compared to 1.47 ± 0.14 mm^2^ (*n* = 6) in the sham group). Therefore surfen impairs the recovery in lesion size when injected 2 days after LPC injection but not at 7 days. To confirm that the day 2 injection of surfen inhibits remyelination of axons rather than inducing axonal loss, ultrastructural examination of axons in the corpus callosum of day 7 lesions was performed. To assess remyelination, 5 mice were examined in each of 3 groups: healthy controls, mice injected with LPC (day 0) followed by vehicle on day 2, and mice injected with LPC (day 0) followed by surfen on day 2. G ratios were calculated from 30 to 40 axons in each mouse, where a G ratio of 1 indicates a denuded axon lacking a myelin sheath. The normal G ratio (in healthy controls) was 0.63 ± 0.01 (mean ± sem, *n* = 193), rising to 0.83 ± 0.01 (*n* = 191) in the LPC-vehicle group and 0.92 ± 0.01 (n = 191) in the LPC-surfen group. Both groups will have a higher G ratio than healthy controls, because remyelination produces thinner myelin sheaths than normal. The percentage of denuded axons (with a G ratio of 1) in the vehicle group was 38.2%, nearly doubling to 73.3% in the surfen group. A significant increase in G ratio was found in both LPC treated groups compared to healthy controls as would be expected, but the G ratio in LPC injected mice co-injected with surfen was significantly higher than the vehicle controls. This indicates that surfen reduced remyelination at day 7, compared to controls injected with LPC alone (Fig. 10). Finally, the lesions were examined for expression of the macrophage-microglial marker Iba-1 and for CSPGs. As expected from the histological sections where macrophages filled the regions of demyelination (see Fig. 8a images), in day 7 mice Iba-1 expression (adjusted for area) was increased in both groups (LPC day 0/vehicle day 2, LPC day 0/surfen day 2), but it was significantly higher in the surfen treated mice compared to vehicle. To confirm this impression, we also counted cells based on DAPI-stained nuclei in the lesions, and found a significant increase in cell counts in the surfen treated group compared to vehicle. CSPG expression also increased in both groups, but was significantly higher in surfen treated mice compared to vehicle (Fig. 11). The ability of surfen to inhibit remyelination may be related to this increase in macrophage-microglial cell density and/or CSPG expression.

## Discussion

Proteoglycans (PGs) represent a potential therapeutic target in MS, because they regulate many aspects of the associated immune responses (which characterize relapsing disease) and remyelination (which by restoring the myelin sheath to axons may prevent or delay progressive disease). To date there are relatively few studies that examine how PG modifying agents like surfen impact disease models of MS. Two reports [12, 14] detail the impact of xyloside and fluorosamine, agents that inhibit the synthesis and secretion of CSPGs. The resulting reduction in tissue CSPGs enhanced remyelination following LPC injection into mouse spinal cord, and fluorosamine treatment also reduced clinical scores in EAE. Other work showed that a sulfated disaccharide derived from CSPGs reduces clinical scores in EAE and also inhibits cytokine secretion by isolated T cells [19, 31]. These studies appear to be contradictory, since they show that disease severity in EAE is reduced both when CSPG synthesis is inhibited, and when components of CSPGs are administered. Surfen has a potentially wider impact than these compounds, because it binds to a variety of GAG side chains on PGs. Its binding results in a fluorescence signal that was strongest for heparin followed by dermatan sulphate, heparan sulphate, and chondroitin sulphate. The binding strength correlates with the degree of sulfation on these GAGs, and binding to heparin was reduced when chemically modified heparins were used that have lower negative charge [20]. Therefore the binding of surfen depends on a charge based ionic interaction, in which positive charged quinoline rings on surfen are thought to bind to negatively charged sulphate and carboxyl groups on the GAG side chains of PGs. The binding of surfen to GAGs appears to cause its molecules to form stacked structures which exhibit an increase in fluorescence, thus providing a useful experimental readout for surfen binding.

What the present study shows is that surfen has a variety of effects on the inflammatory and remyelinating phases of MS, as modeled respectively in mice using EAE and the LPC injection models. Where then does surfen act in these models? In EAE surfen is administered peripherally, so it would have access to peripheral lymph nodes and other sources of circulating immune cells before they enter the CNS. However, surfen is a lipid soluble compound, with a Log *P* value of 2.48 (referring to the logarithm of P, the ratio of solute that dissolves in octanol compared to water). This gives it a high penetration across lipid bilayers, specifically through capillaries in the CNS which form a barrier to water soluble compounds (known as the blood-brain or blood-CNS barrier), but which allow free access to lipid soluble compounds, unless they are specifically extruded by efflux pumps [4]. Therefore it is reasonable to assume that surfen binds to a variety of targets in the CNS during EAE as well as the periphery. Clearly, the direct injection of surfen into the CNS during the LPC model bypasses the blood-brain barrier, and therefore the CNS is the main target in this model. The precise mechanism of action of surfen either on the periphery or in the CNS is unknown, but is likely to result from its direct binding to the GAG side chains of endogenous PGs on a variety of cells. When surfen binds to PGs associated with receptors on the surface of cells, it can prevent these receptors from interacting with a variety of chemokines, cytokines and growth factors. For instance, surfen reduces the ability of Vascular Endothelial Growth Factor to bind to its receptor, which reduces receptor phosphorylation and the resulting increase in dermal vascular permeability [30]. Therefore, surfen may act indirectly by inhibiting other factors from operating. However, surfen can also have direct effects on the functions of immune cells like T cells and macrophages. We have reported that surfen reduces murine T cell proliferation after T cells are stimulated with anti-CD3/CD28 antibody-coated microbeads. When cell surface activation was bypassed, and T cells were activated with phorbyl myristate acetate or ionomycin, surfen had an opposite effect and it increased T cell proliferation, or, at lower doses had no effect [28]. This raises the possibility that the interaction between surfen and cell surface PGs has a direct agonist effect, triggering multiple signaling events in immune and other cell populations that directly alter their function. The first effect is probably steric, in that surfen simply prevents physical interactions from occurring between receptors and their chemokine and growth factor agonists. The second effect may depend on conformational changes in the PGs themselves, so that they act as primary receptors.

Given the wide variety of potential cellular targets for surfen, both in the CNS and in the periphery, its impact on cultured BMDM (Fig. 1) should reflect only part of its mechanisms of action in the animal models. In this regard, there were both similarities and differences when comparing the effect of surfen on LPS-stimulated (activated) BMDM and its actions during EAE. Surfen significantly reduced concentrations of the chemokines CCL2, CCL4 and CCL5 released by lipopolysaccharide (LPS)-stimulated BMDMs (Fig. 1a), while during EAE, reductions were seen for CCL2 and CCL5 but not CCL4 (Fig. 4b). In LPS-stimulated BMDMs, surfen significantly reduced mRNA expression for IL-1β, IL-6 and TNF (Fig. 1b). This differs somewhat from EAE, since surfen reduced mRNA expression for TNF but had no effect on IL-1β or IL-6 (Fig. 5c). Concentrations of IL-6, TNF and IL-10 produced by LPS-stimulated BMDMs were significantly reduced by surfen, while, by contrast, the concentration of IL-1β was significantly increased (Fig. 1c). These effects were not identified during EAE. In LPS-stimulated BMDMs, surfen significantly reduced the mRNA expression of inducible nitric oxide synthase (iNOS), and induced a dose dependent reduction in NO production (Fig. 1d). Changes in iNOS were not detected during EAE (Fig. 5c). It should be emphasized that brain intrinsic glial cells (such as microglia, astrocytes and oligodendrocyte precursor cells) play a vital role in the animal models that were used, including in the deposition by astrocytes of ECM molecules after injury, and work remains to be done in characterizing the impact of surfen on these cell types, both in vitro and in vivo.

During EAE, treatment with surfen resulted in a variety of downstream effects that contributed to a significant reduction in clinical scores (Fig. 2). Clinical scores were recorded for 21 days from induction, and then mice were killed in order to assess the impact of surfen on various parameters, so these results come from the chronic phase of EAE. In general, EAE consists of an early induction phase before clinical signs appear (days 0-7 from inoculation) followed by an acute phase in which scores increase (days 8-16) and a chronic phase where scores reach a plateau (days 17-21) [1]. In the spinal cord of mice with EAE, surfen reduced the number of infiltrating CD4 positive T cells and macrophages (Fig. 3). Surfen treatment also led to significant reductions in the messenger (m)RNA and/or protein expression of the chemokines CCL2, CCL3 and CCL5, with no significant effect on CCL4 (Fig. 4). There are multiple potential mechanism by which surfen could reduce the infiltration of CD4 positive T cells and macrophages into the CNS. Clues come from the reductions in chemokine expression induced by surfen (Fig. 4) and the intriguing albeit preliminary finding that surfen increased numbers and reduced proliferation of T cells in peripheral lymph nodes, although it had no effect on the proportion of CD4 positive T cells in splenic extracts (Additional file 3: Figure S2). This suggests that surfen is acting peripherally as well as centrally, to control T cell migration into the CNS, leading to ‘trapping’ of T cells in the peripheral lymph nodes, as well as reduced chemokine gradients for cellular migration of immune cells from circulating blood into the CNS.

T helper (Th) subsets were also evaluated in the context of EAE (Fig. 5). Broadly speaking, EAE is driven by Th1 and Th17 responses, while Th2 and T-regulatory (Treg) responses are associated with disease resolution. Surfen induced significant reductions in mRNA expression for Th1, Th2 and Treg transcription factors and associated downstream cytokines (Th1 cytokine IL12p40, Th2/Treg cytokine IL-10 and Treg cytokine TGFβ). At the protein level, the Th1 cytokine IL12p40 was also significantly reduced in surfen treated mice, which would tend to reduce EAE disease activity. By contrast, at the protein level, Th2 cytokines were either not changed (IL-5, IL-10, IL-13) or in the case of IL-4 significantly increased. In a previous study, it was shown that IL-4 levels peak during the acute phase of EAE but return to undetectable control levels during the chronic phase [11]. We therefore propose that, in addition to its effects on net cellular migration of T cells and macrophages into the CNS, surfen reduces disease activity through a combination of reduced Th1 responses and enhanced Th2 responses mediated by IL-4, whose levels remain elevated during the chronic phase of surfen treated EAE. If surfen acts in a similar manner to heparanase, an enzyme that inactivates PGs by cleaving heparan sulfate side chains, then our data is consistent with a report in which IL-4 and other Th2 cytokines were induced in ex vivo splenocytes taken from mice treated with heparanase [2]. The persistent elevation in IL-4 may also account for the tendency of surfen to reduce both IL12p40 and the ratio of IL12p40 to IL12p70 (with surfen tending to increase IL12p70 levels). A previous study showed that IL-4 stimulates production of IL-12p70 by dendritic cells and macrophages, while inhibiting IL12-p40 production [10]. The effects of surfen on cytokine expression were more muted in these mice. mRNA expression for Tumor Necrosis Factor (TNF) alone was significantly reduced by surfen, but protein expression was not changed. This likely reflects timing, as many of these cytokines would be past peak expression in the chronic phase of EAE.

Surfen treatment also changed the local expression of mRNA for a variety of PGs (Fig. 6). This data begins to unravel some of the complex effects of different classes of proteoglycan in EAE. mRNA for some of the HSPGs increased significantly during EAE (perlecan, serglycin and syndecan-1) while others were unchanged (syndecan-4, N-deacetylase/N-sulfotransferase-1 and agrin). However, surfen reduced mRNA expression for all six HSPGs, when the EAE groups (vehicle treated and surfen treated) were compared. When clinical scores were plotted as a function of mRNA levels, there were significant positive correlations for the HSPGs serglycin, syndecan-1 and syndecan-4 (Fig. 7). While it is known that HSPGs increase in the brains of patients with MS [27], this data provides evidence linking this expression to deleterious clinical effects. The role that HSPGs play as inhibitors of remyelination is also not well understood, but CSPG expression has been better studied [12, 14, 17]. The implication of these studies is that all CSPGs worsen disease. However, when we examined three CSPGs (neurocan, aggrecan and versican) we found no increases in mRNA expression during EAE, but when EAE groups (vehicle treated and surfen treated) were compared, surfen induced a significant increase in mRNA expression for aggrecan, a decrease for versican, and had no effect on neurocan. Versican expression was previously shown to increase in EAE, and to decrease when EAE was treated with fluorosamine [12], which parallels the effect of surfen. However, alone among the PGs examined, mRNA expression of the CSPG aggrecan had a negative correlation to clinical scores (Fig. 7). Aggrecan is a key component of perineuronal nets involved in synaptic transmission [8], so increased levels of aggrecan induced by surfen may contribute to axonal protection. This lends further nuance to the role of PGs in demyelinating disease of the CNS. While reductions in CSPGs have improved remyelination after administration of LPC [12, 14] our data suggests that in EAE the CSPG aggrecan is protective.

The impact of surfen on remyelination was assessed in the LPC model, and direct injection of surfen into the region of demyelination produced a prolonged delay in remyelination (Figs. 8-10). This inhibitory action was associated with increased local expression of CSPGs in the corpus callosum (Fig. 11), which is similar to the effect of surfen on aggrecan expression during EAE (Fig. 6). We speculate that surfen triggers the synthesis of CSPGs by local cells, either by binding directly to PGs on the cell surface, or by blocking the interaction of regulating factors (cytokines, chemokines, growth factors) with their receptors on these cells. Both astrocytes and macrophages in the CNS have been shown to be sources of CSPGs following LPC injection into the spinal cord [14] and are likely targets for surfen in this model. The increase in Iba-1 labeling as well as cell counts in the surfen group (Fig. 11) suggests that macrophages infiltrate these lesions in larger numbers, and are likely sources for the increase in CSPGs.Other work has shown that LPC injection into the spinal cord increases local expression of versican in the region of demyelination, but aggrecan levels were not changed in nearby grey matter [12]. Since aggrecan is largely confined to perineuronal nets in grey matter, it is unlikely to be found in a white matter tract such as the corpus callosum, and surfen probably increased levels of other CSPGs, with versican a likely candidate. However, in the context of MS plaques, both aggrecan and neurocan were expressed in CNS white matter [27], and both CSPGs inhibited the ability of oligodendrocytes to myelinate axons in tissue culture [17]. Although the two animal models reported here differ in many respects, perhaps the increase in CSPGs induced by surfen has a double effect. Some CSPGs (such as versican) are well known to inhibit remyelination, but others (such as aggrecan) may also be essential as protective factors during inflammation. This raises a dilemma with respect to manipulating PGs in MS using therapeutic agents like surfen, especially if the result is a generalized increase in CSPG expression. This may be therapeutically desirable when CSPGs like aggrecan increase to ameliorate the earlier inflammatory stages of MS, but therapeutically problematic when other CSPGs such as versican increase, that inhibit remyelination and repair in later phases of the disease.

## Additional files

Additional file 1: Table S1. List of qRT-PCR primers used to determine mRNA expression (TIFF 1810 kb)

Additional file 2: Figure. S1. **a,b**. Surfen affects viability of cultured bone marrow derived macrophages (BMDMs) at higher doses (a as assessed by 7-ADD staining, b as assessed by MTT assay, doses indicated). c. Surfen (5 μM) binding to BMDMs is reduced by co-application of heparitinase-III and chondroitinase ABC, alone or in combination. Data is shown as mean ± SEM from 4 independent experiments. Significance compares surfen with vehicle unless otherwise indicated by cross bars (* = *P* < 0.05) (TIFF 1367 kb)

Additional file 3: Figure S2. During EAE, surfen increases lymph node cell count, but reduces proliferation in extracted CD4 positive T cells. Superficial cervical, axillary, brachial and inguinal lymph nodes were pooled from individual mice (n shown). Total cells were counted (a) and then isolated T cells were stimulated in vivo with anti-CD3, anti CD-28 T cell expander beads for 24 h, and proliferation assessed by Oregon Green staining (b). Spleens were also homogenized (n indicates number of mice, with one spleen per mouse) and cells stained with antibodies directed against surface markers and then analyzed by flow cytometry. The percentage of CD4 positive T cells among the extracts is shown (c) along with the gating strategy (d). Data compare EAE treated with vehicle (EAE-V) or surfen (EAE-S), and is shown as mean ± SEM. Significance compares surfen with vehicle (* = *P* < 0.05) (TIFF 1933 kb)

Additional file 4: Figure S3. Surfen injected at the same time as LPC has no effect on lesion size 7 days later. Left sided linear graphic shows treatment schedule, while right hand panel shows data for each group as well as representative images of lesions in the corpus callosum. Data is shown as mean ± SEM. Scale bars = 200 μm (TIFF 6018 kb)

## References

[1] Barthelmes J, Tafferner N, Kurz J, de Bruin N, Parnham MJ, Geisslinger G et al (2016) Induction of experimental autoimmune encephalomyelitis in mice and evaluation of the disease-dependent distribution of immune cells in various tissues. J Vis Exp Doi:10.3791/53933 10.3791/53933 PMC4942059 27214391

[2] Bitan M, Weiss L, Reibstein I, Zeira M, Fellig Y, Slavin S (2010). Heparanase upregulates Th2 cytokines, ameliorating experimental autoimmune encephalitis. Mol Immunol.

[3] Bjartmar C, Trapp B (2001). Axonal and neuronal degeneration in multiple sclerosis: mechanisms and functional consequences. Curr Opin Neurol.

[4] Bodor N, Buchwald P (1999). Recent advances in the brain targeting of neuropharmaceuticals by chemical delivery systems. Adv Drug Deliv Rev.

[5] Dyck SM, Karimi-Abdolrezaee S (2015). Chondroitin sulfate proteoglycans: key modulators in the developing and pathologic central nervous system. Exp Neurol.

[6] Fiander MDJ, Stifani N, Nichols M, Akay T, Robertson GS (2017). Kinematic gait parameters are highly sensitive measures of motor deficits and spinal cord injury in mice subjected to experimental autoimmune encephalomyelitis. Behav Brain Res.

[7] Foote AK, Blakemore WF (2005). Inflammation stimulates remyelination in areas of chronic demyelination. Brain.

[8] Giamanco KA, Morawski M, Matthews RT (2010). Perineuronal net formation and structure in aggrecan knockout mice. Neuroscience.

[9] Jeffrey ND, Blakemore WF (1995). Remyelination of mouse spinal cord axons demyelinated by local injection of lysolecithin. J Neurocytol.

[10] Kaliński P, Smits HH, Schuitemaker JH, Vieira PL, van Eijk M, de Jong EC (2000). IL-4 is a mediator of IL-12p70 induction by human Th2 cells: reversal of polarized Th2 phenotype by dendritic cells. J Immunol.

[11] Kennedy MK, Torrance DS, Picha KS, Mohler KM (1992). Analysis of cytokine mRNA expression in the central nervous system of mice with experimental autoimmune encephalomyelitis reveals that IL-10 mRNA expression correlates with recovery. J Immunol.

[12] Keough MB, Rogers JA, Zhang P, Jensen SK, Stephenson EL, Chen T (2016). An inhibitor of chondroitin sulfate proteoglycan synthesis promotes central nervous system remyelination. Nat Commun.

[13] Kotter MR, Zhao C, van Rooijen N, Franklin RJM (2005). Macrophage-depletion induced impairment of experimental CNS remyelination is associated with a reduced oligodendrocyte precursor cell response and altered growth factor expression. Neurobiol Dis.

[14] Lau LW, Keough MB, Haylock-Jacobs S, Cua R, Doring A, Sloka S (2012). Chondroitin sulphate proteoglycans in demyelinated lesions impair remyelination. Ann Neurol.

[15] Livak KJ, Schmittgen TD (2001). Analysis of relative gene expression data using real-time quantitative PCR and the 2(−Delta Delta C(T)) method. Methods.

[16] Miron VE, Boyd A, Zhao J-W, Yuen TJ, Ruckh JM, Shadrach JL (2013). M2 microglia and macrophages drive oligodendrocyte differentiation during CNS remyelination. Nat Neurosci.

[17] Pendleton JC, Shamblott MJ, Gary DS, Belegu V, Hurtado A, Malone ML, McDonald JW (2013). Chondroitin sulfate proteoglycans inhibit oligodendrocyte myelination through PTPσ. Exp Neurol.

[18] Raine CS, Wu E (1993). Multiple sclerosis: remyelination in acute lesions. J Neuropath Exp Neurol.

[19] Rolls A, Cahalon L, Bakalash S, Avidan H, Lider O, Schwartz M (2006). A sulfated disaccharide derived from chondroitin sulfate proteoglycan protects against inflammation-associated neurodegeneration. FASEB J.

[20] Schuksz M, Fuster MM, Brown JR, Crawford BE, Ditto DP, Lawrence R (2008). Surfen, a small molecule antagonist of heparan sulfate. PNAS.

[21] Setzu A, Lathia JD, Zhao C, Wells K, Rao MS, Ffrench-Constant C (2006). Inflammation stimulates myelination by transplanted oligodendrocyte precursor cells. Glia.

[22] Shute J (2012). Glycosaminoglycan and chemokine/growth factor interactions. Handbook Exper Pharmacol.

[23] Sobel RA, Ahmed AB (2001). White matter extracellular matrix chondroitin sulphate/dermatan sulphate proteoglycans in multiple sclerosis. J Neuropath Exp Neurol.

[24] Stromnes IM, Goverman JM (2006). Active induction of experimental allergic encephalomyelitis. Nat Protoc.

[25] Stys PK, Zamponi GW, van Minnen J, Geurts JG (2012). Will the real multiple sclerosis please stand up?. Nat Rev Neurosci.

[26] Taylor S, Wakem M, Dijkman G, Alsarraj M, Nguyen M (2010). A practical approach to RT-qPCR-publishing data that conform to the MIQE guidelines. Methods.

[27] van Horssen J, Bö L, Dijkstra CD, de Vries HE (2006). Extensive extracellular matrix depositions in active multiple sclerosis lesions. Neurobiol Dis.

[28] Warford J, Doucette CD, Hoskin DW, Easton AS (2014). Murine T cell activation is regulated by surfen (*bis*-2-methyl-4-amino-quinolyl-6-carbamide). Biochem Biophys Res Commun.

[29] Woodruff RH, Franklin RJM (1999). Demyelination and remyelination of the caudal cerebellar peduncle of adult rats following stereotactic injections of lysolecithin, ethidium bromide and complement/anti-galactocerebroside: a comparative study. Glia.

[30] Xu D, Fuster MM, Lawrence R (2011). Esko JD (2011) Heparan sulfate regulates VEGF165- and VEGF121-mediated vascular hyperpermeability. J Biol Chem.

[31] Zhou J, Nagarkatti P, Zhong Y, Nagarkatti M (2010). Immune modulation by chondroitin sulfate and its degraded disaccharide product in the development of an experimental model of multiple sclerosis. J Neuroimmunol.

[32] Zimmerman DR, Dours-Zimmermann MT (2008). Extracellular matrix of the central nervous system: from neglect to challenge. Histochem Cell Biol.

