# Supplementary figures and images for: Surfen, a proteoglycan binding agent, reduces inflammation but inhibits remyelination in murine models of Multiple Sclerosis
Source: Acta Neuropathol Commun. 2018 Jan 4;6:4. doi: 10.1186/s40478-017-0506-9 (PMC5755315; doi:10.1186/s40478-017-0506-9)

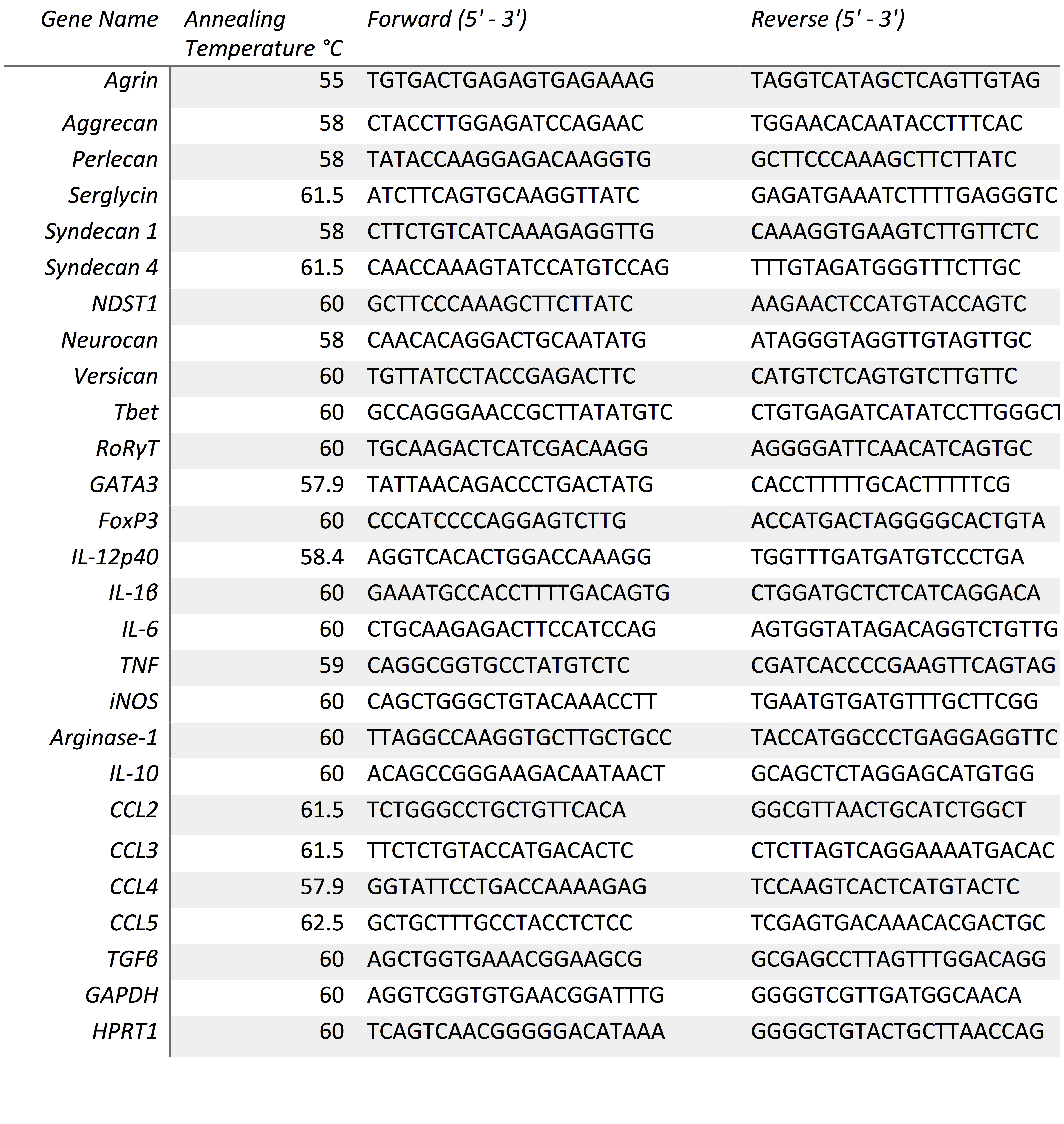

Supplement: Supplementary file 1 — List of qRT-PCR primers used to determine mRNA expression (TIFF 1810 kb) [file 40478_2017_506_MOESM1_ESM.tiff]

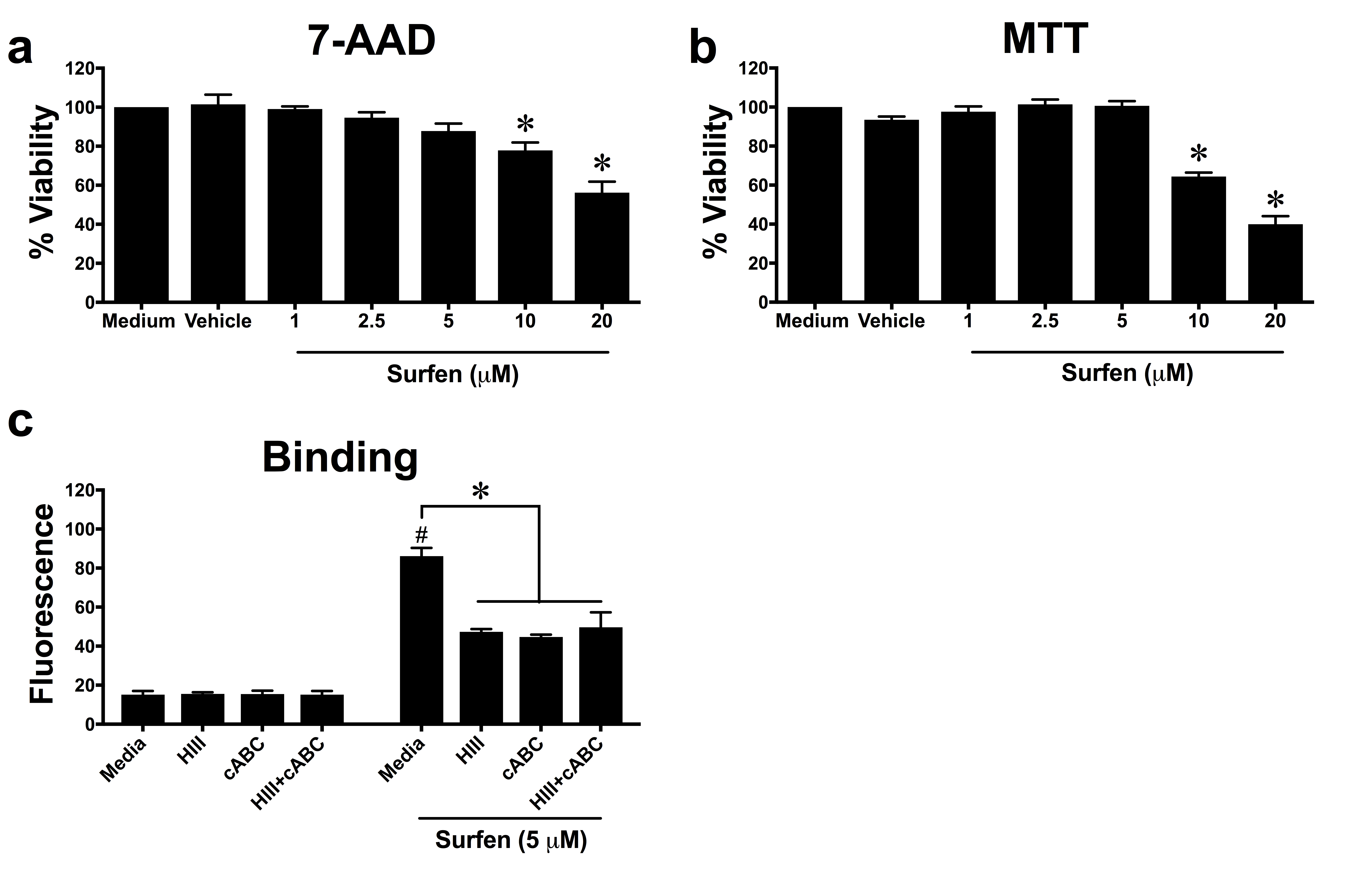

Supplement: Supplementary file 2 — a,b. Surfen affects viability of cultured bone marrow derived macrophages (BMDMs) at higher doses (a as assessed by 7-ADD staining, b as assessed by MTT assay, doses indicated). c. Surfen (5 μM) binding to BMDMs is reduced by co-application of heparitinase-III and chondroitinase ABC, alone or in combination. Data is shown as mean ± SEM from 4 independent experiments. Significance compares surfen with vehicle unless otherwise indicated by cross bars (* = P < 0.05) (TIFF 1367 kb) [file 40478_2017_506_MOESM2_ESM.tiff]

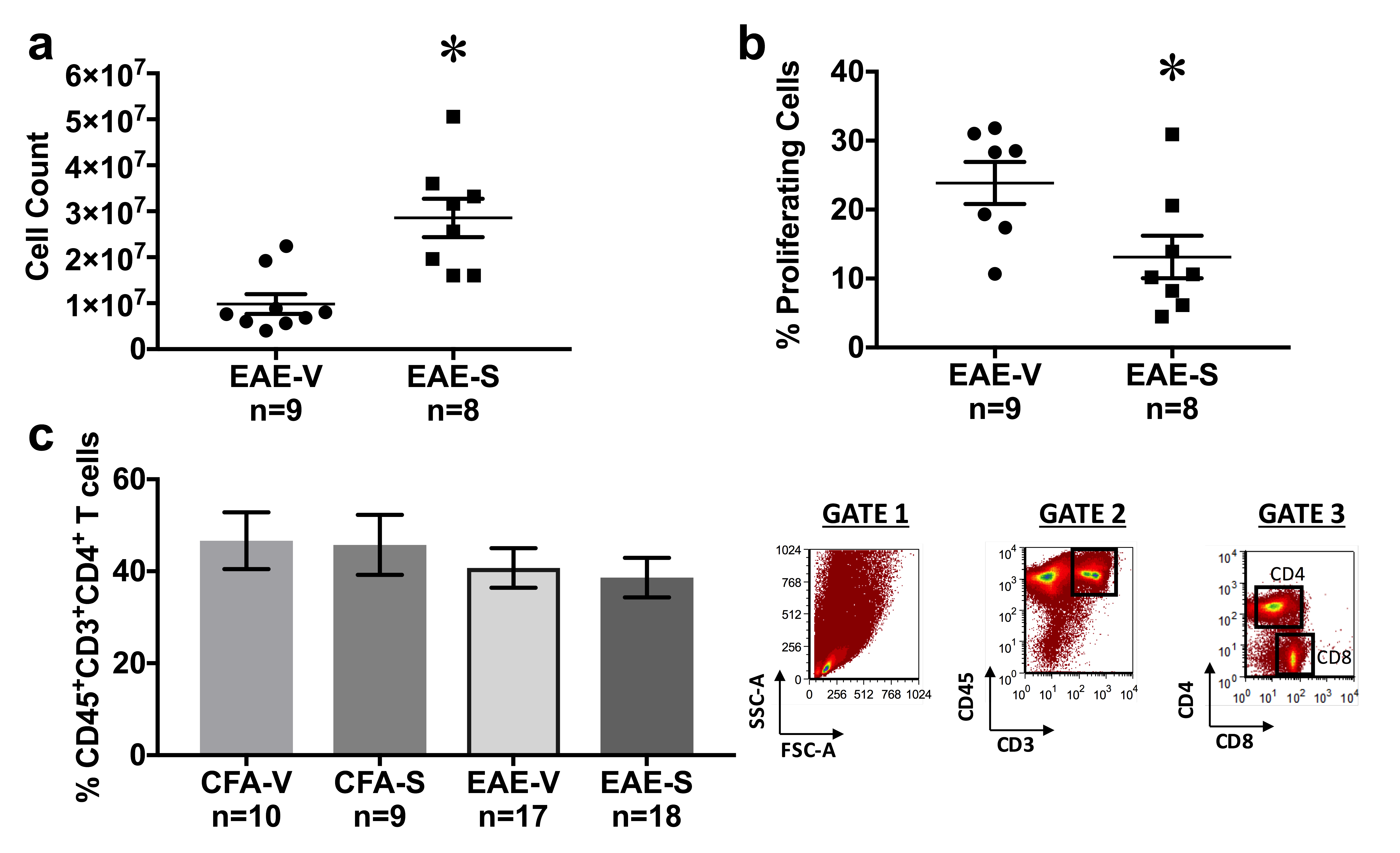

Supplement: Supplementary file 3 — During EAE, surfen increases lymph node cell count, but reduces proliferation in extracted CD4 positive T cells. Superficial cervical, axillary, brachial and inguinal lymph nodes were pooled from individual mice (n shown). Total cells were counted (a) and then isolated T cells were stimulated in vivo with anti-CD3, anti CD-28 T cell expander beads for 24 h, and proliferation assessed by Oregon Green staining (b). Spleens were also homogenized (n indicates number of mice, with one spleen per mouse) and cells stained with antibodies directed against surface markers and then analyzed by flow cytometry. The percentage of CD4 positive T cells among the extracts is shown (c) along with the gating strategy (d). Data compare EAE treated with vehicle (EAE-V) or surfen (EAE-S), and is shown as mean ± SEM. Significance compares surfen with vehicle (* = P < 0.05) (TIFF 1933 kb) [file 40478_2017_506_MOESM3_ESM.tiff]

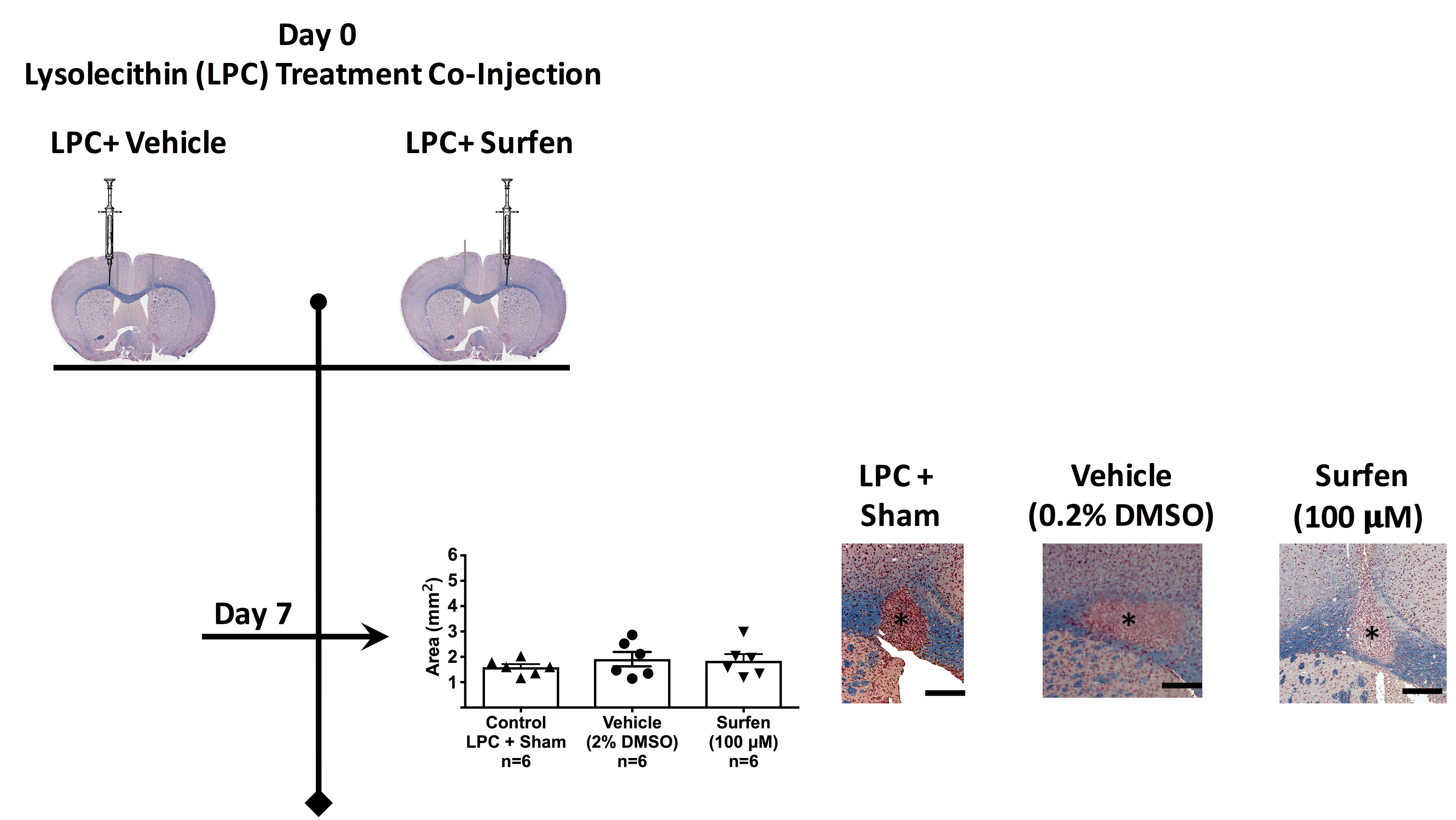

Supplement: Supplementary file 4 — Surfen injected at the same time as LPC has no effect on lesion size 7 days later. Left sided linear graphic shows treatment schedule, while right hand panel shows data for each group as well as representative images of lesions in the corpus callosum. Data is shown as mean ± SEM. Scale bars = 200 μm (TIFF 6018 kb) [file 40478_2017_506_MOESM4_ESM.tiff]
